# Preventative and therapeutic potential of animal milk components against COVID‐19: A comprehensive review

**DOI:** 10.1002/fsn3.3314

**Published:** 2023-03-23

**Authors:** Parminder Singh, Roberto Hernandez‐Rauda, Oscar Peña‐Rodas

**Affiliations:** ^1^ Department of Animal Husbandry Amritsar Government of Punjab Amritsar India; ^2^ Laboratorio de Inocuidad de Alimentos Universidad Doctor Andres Bello San Salvador El Salvador, América Central

**Keywords:** animal milk, anti‐inflammatory, antivirals, COVID‐19, immunomodulators, milk components

## Abstract

The global pandemic of COVID‐19 is considered one of the most catastrophic events on earth. During the pandemic, food ingredients may play crucial roles in preventing infectious diseases and sustaining people's general health and well‐being. Animal milk acts as a super food since it has the capacity to minimize the occurrence of viral infections due to inherent antiviral properties of its ingredients. SARS‐CoV‐2 virus infection can be prevented by immune‐enhancing and antiviral properties of caseins, α‐lactalbumin, β‐lactoglobulin, mucin, lactoferrin, lysozyme, lactoperoxidase, oligosaccharides, glycosaminoglycans, and glycerol monolaurate. Some of the milk proteins (i.e., lactoferrin) may work synergistically with antiviral medications (e.g., remdesivir), and enhance the effectiveness of treatment in this disease. Cytokine storm during COVID‐19 can be managed by casein hydrolyzates, lactoferrin, lysozyme, and lactoperoxidase. Thrombus formation can be prevented by casoplatelins as these can inhibit human platelet aggregation. Milk vitamins (i.e., A, D, E, and B complexes) and minerals (i.e., Ca, P, Mg, Zn, and Se) can have significantly positive effects on boosting the immunity and health status of individuals. In addition, certain vitamins and minerals can also act as antioxidants, anti‐inflammatory, and antivirals. Thus, the overall effect of milk might be a result of synergistic antiviral effects and host immunomodulator activities from multiple components. Due to multiple overlapping functions of milk ingredients, they can play vital and synergistic roles in prevention as well as supportive agents during principle therapy of COVID‐19.

## Graphical abstract

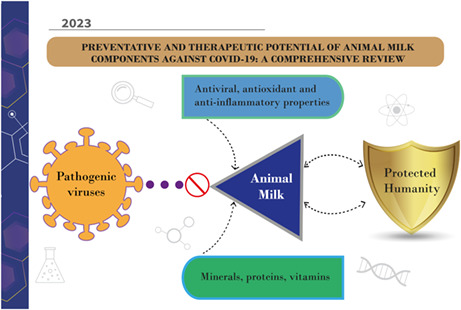

## 1 INTRODUCTION

Due to rapid globalization and human activities, a number of emerging and reemerging viral diseases, such as pandemic influenza H1N1, highly pathogenic avian influenza H5N1, Zika virus, Ebola virus, chikungunya virus, Lassa virus, Japanese encephalitis virus, Kyasanur forest disease virus, Nipah virus, coronavirus (CoV) diseases, that is, the Middle East respiratory syndrome‐related CoV (MERS‐CoV) and severe acute respiratory syndrome CoV (SARS‐CoV), have happened in the past (Liu et al., 2016). Similar to this, in late December 2019, suddenly a number of severe infectious cases of pneumonia with flu‐like symptoms were noticed in wet seafood market of Wuhan, China (Wu et al., 2020). Later on, World Health Organization (WHO) identified the disease as CoV disease 2019 (COVID‐19) caused by severe acute respiratory syndrome CoV‐2 (SARS‐CoV‐2). Afterward, the disease spread very quickly as a pandemic on the entire planet due to its high virulence and infectivity. Moreover, the disease adversely affected public health, economy, and global biosafety system of various countries. As per the latest global situation report of WHO, there were 652 million confirmed cases of COVID‐19, resulting in almost 6.66 million fatalities worldwide as of December 23, 2022 (WHO, 2022a). Due to sudden surge of COVID‐19 cases in different countries, on December 21, 2022, Dr. Tedros Adhanom Ghebreyesus (WHO chief) warned all the countries regarding the unprecedented serious illness caused by omicron subvariant BF.7 of SARS‐CoV‐2, and advised to follow COVID appropriate behavior along with necessary measures to overcome the impending outbreak (WHO, 2022b). Although the injectable vaccine has been made available in many countries as a preventive measure to manage the outbreak, there are certain concerns about the safety and efficacy of vaccines as the pandemic is still ongoing. For therapeutic purpose, FDA has approved emergency use of certain antiviral drugs such as remdesivir, molnupiravir, and Paxlovid (combination of nirmatrelvir and ritonavir) for treatment of individuals who are more likely to get a serious COVID‐19 condition that could result in hospitalization and/or death (Joyce et al., 2022). However, there is a need of natural, economical, and orally bioavailable immunomodulators and antivirals that can be given in outpatient settings to reduce COVID‐19‐related hospitalizations.

Foods and/or their ingredients have been reported to act as antivirals, immunomodulators, and anti‐inflammatory agents due to their inherent neutraceutical properties. Among these foods is animal milk (AM) which is usually referred to as a distinctive lacteal secretion endowed with various bioactive ingredients including proteins, lipids, oligosaccharides, vitamins and minerals. These ingredients have ability to influence a wide range of physiological functions including growth, development, and other neutraceutical roles required for the maintaining the general health of individuals (Gallo et al., 2022). In the present review, an attempt has been made to highlight the immunomodulatory, antiviral, anti‐inflammatory, and other therapeutic properties of various AM ingredients in context of viral diseases with special emphasis on COVID‐19.

## 2 MILK PROTEINS

Milk proteins can be broadly classified into two categories, that is, caseins (CNs) and whey proteins (WPs). The concentration of some important CNs and WPs in AM and colostrum of different species are mentioned in Table 1.

**TABLE 1:** Concentration of various proteins in milk of different species.

Type of milk proteins	Bovine	Equine	Caprine	Ovine	Camel
Caseins					
α_S1_‐casein (g/L)	10.4–13 (CMM), 14.4–18 (BMM)	2.5 (MM)	1.344 (MM)	3.149 (MM)	2.4–10.3 (MM)
α_S2_‐casein (g/L)	2.6–3.4 (CMM), 2.2–2.8 (BMM)	0.20 (MM)	4.608 (MM)	10.716 (MM)	0.3–3.9 (MM)
Whey proteins					
α‐lactalbumin (g/L)	1–3 (Col), 1.4 (MM)	2.37 (MM)	1.98 (MM)	1.16 (MM)	0.3–2.9 (MM)
β‐lactoglobulin (g/L)	6–14 (Col), 3 (MM)	2.55 (MM)	5.61 (MM)	6.57 (MM)	None
IgG (total) (g/L)	32–212 (Col), 0.72 (MM)	0.39 (MM)	4.8–75 (Col), 0.70 (MM)	6.2–65.4 (Col), 0.55 (MM)	4.75–132.5 (Col), 1.64 (MM)
Serum albumin (g/L)	1.3 (Col), 0.30 (MM)	0.37 (MM)	2.97 ± 2.46 (Col), 0.26–0.30 (MM)	0.49–0.55 (MM)	0.46 (MM)
Lactoferrin (mg/mL)	1.5–5 (Col), 0.02–0.75 (MM)	0.0061–0.0621 (Col), 0.58 (MM)	0.39 (Col), 0.06 (MM)	0.74 (Col), 0.12 (MM)	0.59–5.10 (Col), 0.18–2.48 (MM)
Lysozyme (mg/L)	0.3–0.8 (Col), 0.1 (MM)	0.87 (MM)	0.25 (MM)	0.20 (MM)	0.15 (MM)
Lactoperoxidase activity (Units/mL)	1.4 (CMM), 0.9 (BMM)	Nil (MMM), 0.0048 ± 0.35 (DSM)	1.55 (MM)	0.14–2.38 (MM)	2.23 ± 0.01 (MM)

Abbreviations: BMM, buffalo mature milk; Col, colostrum; CMM, cow mature milk; DSM, donkey's skimmed milk; MM, mature milk; MMM, mare's mature milk.

Source: Balthazar et al. (2017); Benkerroum (2008); Brumini (2013); El‐Agamy and Nawar (2000); El‐Hatmi et al. (2007); Jahan et al. (2020); Kessler et al. (2019); Khan et al. (2019); Konuspayeva et al. (2007); Konuspayeva (2020); Li et al. (2019); Lonnerdal (2014); Marnila and Korhonen (2002); McGrath et al. (2016); Mohamed et al. (2020); Navarro et al. (2018); Olaniyan (2007); Prosser (2021); Quinn (2021); Rieland et al. (1998); Seifu et al. (2005); Singh et al. (2017); Vincenzetti et al. (2012); Wheeler et al. (2007).

### 2.1 Caseins

Caseins represent one of the most heterogenous classes of AM proteins, comprising three sub‐forms—α, β, and κ. The respective percentages of α, β, and κ‐CN in bovine milk are 45, 30, and 15%. Of all the forms, the most calcium‐sensitive form is α_S2_. Among them, κ‐CN (15% of all casein fractions in bovine milk) is a phosphoglycoprotein, which is unique with three *o*‐glycosylation sites (Singh et al., 2018). Moreover, the glycan structure of κ‐CN changes with the transformation from colostrum to mature AM (Saito et al., 1981).

#### 2.1.1 Immunomodulator properties

While cell‐mediated immunity helps to eliminate the COVID‐19 viral infection of cells, innate and humoral adaptive immunity focuses on preventing this infection from happening (Abdulamir & Hafidh, 2020). This is particularly important for weak, old, and comorbid patients. Various bioactive peptides derived from α_S1_‐CN have been reported to stimulate both phagocytosis and antibody formation (Jolles et al., 1992; Meisel, 1997). For example, isracidin, a biologically active peptide generated from chymosin treatment of α_S1_‐CN, possesses strong immunomodulating properties such as stimulation of lymphocytes proliferation, natural killer (NK) cell activity, and neutrophil locomotion (Elitsur & Luk, 1991; Migliore‐Samour & Jolles, 1988). Isracidin is commonly found in fermented AM products like yogurt and cheese. β‐CN is a calcium‐sensitive phosphoprotein that makes up about 40% and one‐third of the total CN and total protein content of bovine milk, respectively (Daniloski et al., 2021). According to a research study, bovine β‐CN (1–28) stimulated the proliferation of human B cells, monocytes, and T cells and activated the production of IgA from human B cell lines (Kawahara et al., 2004). This might be mediated through μ‐receptors present on the cell membranes of lymphocytes. Bovine β‐CN is also reported to exhibit selective impact on both native and adaptive immune responses in ruminants (Wong et al., 1996). Another study revealed that a bovine β‐CN peptide “Pro‐GIy‐Pro‐Ile‐Pro‐Asn” was immunologically analogous to human β‐CN hexapeptide “Val‐Glu‐Pro‐lle‐Pro‐Tyr” which had capability of stimulating phagocytosis in mice (Migliore‐Samour & Jolles, 1988). Numerous opioid peptides known as “β‐casomorphins (BCMs)” are produced as a result of the hydrolytic cleavage of CNs in the body. These BCM peptides (BCM‐4, BCM‐5, BCM‐6, BCM‐7, BCM‐8, BCM‐9, BCM‐11, BCM‐13, and BCM‐21) as well as derivatives created by their further hydrolysis play a variety of physiological and neutraceutical roles by binding to opioid receptors found both in neuronal and nonneuronal tissues (Sobczak et al., 2014). They share the same N‐terminal sequence and initial three amino acids in their structure “Tyr‐Pro‐Phe,” and promote antibody synthesis and phagocytosis (Fiat & Jollès, 1989; Jolles et al., 1981). On passing through epithelial lining of gut, they enhance the enzymatic action and expression of dipeptidyl peptidase IV (DPP‐4, an enzyme produced by enterocytes), which in turn activates “Th2 immunological pathway” and nonspecific inflammatory response (Daniloski et al., 2021). Moreover, diminished performance of DPP‐4 is typically linked to a weakened immune system (Jarmołowska et al., 2019; Uematsu et al., 1996). Thus, BCMs mediate endorphin‐like activity on the development of T lymphocyte function and cellular immunity via μ‐receptors. Besides acting as a potent immunomodulator, BCM‐7 enhances the production of mucin which helps to defend the gut against enteric infections (Asledottir et al., 2019; Trompette et al., 2003). Caseinomacropeptide (CMP) is released into whey after chymosin digestion of κ‐CN during the preparation of cheese. The remaining part of κ‐CN gets precipitated into cheese curd and is known as para‐κ‐CN. The immunomodulating effects of both intact CMP as well its peptides depend upon the polypeptide portion of CMP and the presence of sialic acid (Li & Mine, 2004; Otani et al., 1995). According to Sutas et al. (1996), it has been reported that some κ‐CN peptides (released after its hydrolytic cleavage by trypsin) stimulated the mitogen‐induced proliferation of human lymphocytes. Similarly, bovine para‐κ‐CN (1–105 regions) has been reported to stimulate antibody formation and phagocytic activity of murine and human macrophages in vitro (Jolles et al., 1988; Jolles & Migliore‐Samour, 1986). Another peptide Tyr‐Gly (383–389) derived from κ‐CN enhanced cellular proliferation of human peripheral blood lymphocytes (HPBL) activated with concanavalin A in vivo (Kayser & Meisel, 1996; Meisel, 1997). Bovine glycomacropeptide (κ‐CN derivative) also exhibits immunomodulatory (immunostimulative and immunosuppressive) properties. It may cause sialic acid‐dependent inhibition of murine lymphocyte (T and B) proliferation (Otani et al., 1995), as well as may encourage monocytes to upregulate the IL‐1 (interleukin‐1) receptor antagonist, an anti‐inflammatory molecule (Monnai & Otani, 1997). These findings suggest that CNs may be used very well in fight against COVID‐19 but further studies are required.

#### 2.1.2 Antiviral properties

Caseins and their fragments have been reported to exert antiviral activities against some pathogenic viruses in humans (Table 2). As reported in these in vitro studies, different complex mechanisms are responsible for their antiviral activity. Rubin et al. (2021) reported that antiviral potential of goat milk was greater than bovine milk. The authors reported antiviral activity of goat milk CN (GMC) against Coxsackievirus A9 and SARS‐CoV‐2 pseudovirus. Intriguingly, GMC exhibited stronger antiviral impact when it was preincubated with the virus than when it was supplemented with a cell–virus blend. The effect of GMC on SARS‐CoV‐2 pseudovirus was studied by computing fluorescence to assess if the virus enters the cell, and a greater than 75% suppression of viral entry was observed. This speculative process has to be investigated for COVID‐19 further in the in vitro experiments. Dash and Jaganmohan (2022) isolated a therapeutic peptide “RYLGY” from cold plasma treated α_S1_‐CN of cow milk, which may disrupt the attachment between RBD of the SARS‐CoV‐2 spike protein and ACE2 receptors of the cell membrane, preventing the virus from entering cells. An improved binding affinity and electrostatic interactions were reported between the peptide and ACE2‐RBD complex as demonstrated by in silico docking studies. To fully comprehend the impact of casein peptides for efficient targets of COVID‐19, additional in vivo research is mandatory.

**TABLE 2:** Antiviral roles of caseins and their derivatives against some important pathogenic viruses of humans.

S No.	Type of casein	Name of virus	In vitro mechanism of action	References
1.	Bovine CN (chemically modified)	HIV‐1	Preventing HIV‐1 infection by blocking the binding between the envelope glycoproteins of HIV‐1 (gp 120) and CD4 cell receptors	Neurath et al. (1995)
2.	Bovine α_S1_‐CN (sialylated)	Influenza A virus	Inhibition of virus attachment to cell surface receptors	Yu et al. (2018)
3.	Bovine α_S2_‐CN (3 HP)	HIV‐1	The random coil of a negatively charged polypeptide could be the actual antiviral molecule	Berkhout et al. (1997)
4.	Camel milk CN hydrolyzates	Coxsackie virus B6	Blocking the virus entry into host cells by allowing entry of hydrophobic inhibitory molecules in the hydrophobic binding cavities of the viral surface	Abbes et al. (2021)
5.	Goat milk CN	HSV‐1	Inhibition of replication by interacting with viral envelope	Rubin et al. (2021)
6.	Donkey milk CN	Echovirus type 5	Inhibition of replication	Brumini et al. (2013)
7.	bGMP (κ‐CN derivative)	Human rotavirus	Direct binding to virus particles via glycan residues	Inagaki et al. (2014)
8.	bGMP (κ‐CN derivative)	Influenza virus A and B	Preventing virus attachment to host cells and inhibition of hemagglutination activity	Kawasaki et al. (1993)
9.	bGMP (κ‐CN derivative)	Epstein–Barr virus	Preventing morphological changes in peripheral blood lymphocytes	Dosako et al. (1992)

Abbreviations: bGMP, bovine glycomacropeptide; CN, casein; HIV, human immunodeficiency virus; HSV, herpes simplex virus.

#### 2.1.3 Antioxidant and anti‐inflammatory properties

Oxidative stress and inflammation occur concomitantly and act as key players in many disease conditions including COVID‐19 (Delgado‐Roche & Mesta, 2020). So far a number of scientific studies have reported the antioxidant (free radical scavenging and metal chelating activities) and anti‐inflammatory activities of CN‐derived peptides (Altmann et al., 2016; Bamdad et al., 2017; Chen et al., 2022; Li, Cheng, et al., 2017; Mukhopadhya et al., 2015; Oh et al., 2017; Shi & Zhao, 2022; Yoo et al., 2021). Mao et al. (2011) prepared yak milk CN hydrolyzates with enzyme alcalase and observed their significant free radical scavenging activities in terms of 2,2‐diphenylpicrylhydrazyl, superoxide, and hydrogen peroxide (H_2_O_2_). They also documented decrease in the production of nitric oxide (NO), and other proinflammatory cytokines such as IL‐6, TNF‐α (tumor necrosis factor‐α), and IL‐1β in a concentration‐dependent manner in lipopolysaccharide (LPS)‐stimulated murine peritoneal macrophages. Another study documented the anti‐inflammatory role of bovine milk sodium caseinate hydrolyzates in both in vitro and ex vivo colon models. The study proved reduction in concentrations of IL‐8 and other proinflammatory cytokines (IL1‐α, IL1‐β, IL‐8, TGF‐β (transforming growth factor‐β), and IL‐10) in in vitro and ex vivo systems, respectively (Mukhopadhya et al., 2014). Similarly, a bovine tryptic β‐CN hydrolyzate showed anti‐inflammatory effect by inhibiting NFκB (a proinflammatory transcription factor of several genes) in vitro (Malinowski et al., 2014). Various CN‐derived peptides, namely tripeptide—“LLY” (Sowmya et al., 2018), buffalo milk CN‐derived hexapeptide—“YFYPQL” (Sowmya et al., 2019a), and decapeptide—“YQEPVLGPVR” (Sowmya et al., 2019b), displayed anti‐inflammatory effect by reducing proliferation of murine splenocytes, modulating the production of inflammatory cytokines (IFN‐γ (interferon‐γ), IL‐10, and TGF‐β), and enhancing the phagocytosis of peritoneal macrophages under ex vivo conditions. However, their antioxidative properties were attributed to various factors, that is, protection against H_2_O_2_‐induced oxidative cell death, reduction in generation of reactive oxygen species (ROS), and enhanced activities of antioxidative enzymes (catalase) by stimulating the NRF‐2 (nuclear response factor‐2) stress signaling pathway under cellular (Caco‐2) assessment. Among these three peptides, tripeptide also showed potent antioxidative and anti‐inflammatory effects in mice. As an anti‐inflammatory molecule, a CN peptide “Gln‐Glu‐Pro‐Val‐Leu” was reported to regulate the production of NO and cytokines (IL‐4, IL‐10, IFN‐γ, and TNF‐α) under in vivo conditions (Jiehui et al., 2014). Thus, CNs may play important role both in the prevention of oxidative stress and inflammation‐related disorders such as COVID‐19.

#### 2.1.4 Antithrombotic properties

In COVID‐19, thrombus formation can occur either during the disease or after COVID‐19 vaccination due to binding of fibrinogen with platelet membranes, which in turn forms the effective link between the platelets and causes platelet aggregation (Iba et al., 2021; Waggiallah, 2021). According to a study, it has been reported that κ‐CN macropeptides of bovine, ovine, and caprine origin exhibited in vitro antithrombotic properties due to inhibition of human platelet aggregation by casoplatelin (106–116 regions of κ‐CN) (Manso et al., 2002). This might be due to structural homology and molecular similarity of casoplatelin with the human fibrinogen γ‐chain (400–411 fragment) which prevents binding between the latter and the platelet membrane (Ren et al., 2016). Furthermore, tryptic hydrolyzates derived from casoplatelin of different species have been shown to inhibit platelet aggregation in vitro (Leonil & Molle, 1991). Caseinoglycopeptide residues, that is, 106–169, 106–116, and 112–116 regions produced from cow κ‐CN, showed both in vitro and in vivo antiplatelet aggregating activities (dit Sollier et al., 1996). The study also revealed significant antithrombotic activity of human, bovine, and ovine caseinoglycopeptides when demonstrated in a guinea pig model of arterial thrombosis caused by laser‐induced intimal damage. Some studies have reported the presence of active fragments of κ‐CN in the circulatory system of rats and humans (Chabance et al., 1998; Fosset et al., 2002). This indicates the evidence for generation of these peptides after complete digestion of AM or yogurt, in addition to the inability of active sequences produced in vitro or in vivo to withstand subsequent digestion. Caseinoglycopeptide residues (106–171) obtained from sheep κ‐CN inhibited collagen and thrombin‐induced platelet aggregation in a dose‐dependent style (Qian et al., 1995). Three peptide residues (112–116, 163–171, and 165–171 regions) fully prevented thrombin‐induced platelet aggregation. Thus, CNs may act as good clinical candidates as antithrombotic agents during the treatment of COVID‐19.

### 2.2 Whey proteins

#### 2.2.1 Immunomodulator properties

Various WPs such as whole WPs, lactoferrin (LF), lactoperoxidase (LPO), milk growth factors, IgGs, and/or their enzymatic fractions (trypsin/chymotrypsin) act as crucial players in immunomodulation (Cross & Gill, 2000). Compared to CNs, WPs have a significant excess of cysteine. For glutathione (GSH) production, which is essential for lymphocyte proliferation, dietary cysteine is thought to be a rate‐limiting substrate (Phelan et al., 2020). Moreover, it was documented in a study that stimulation of host humoral immune response was linked with greater and longer‐lasting release of splenic GSH during the antigen‐driven clonal proliferation of the lymphocytes in mice receiving WPs as a part of the diet (Bounous et al., 1989). In vitro experiments proved that bovine milk proteins also have the capacity to boost neutrophil oxidative responses, primarily in heterologous species (Wong, Seow, et al., 1997).

Milk proteins such as whey protein concentrates (WPCs) are typically regarded as immunostimulatory agents, despite the fact that different whey protein isolates (WPIs) or their fractions have been found to have vastly disparate effects on immune function (Gill & Rutherfurd, 1998; Knowles & Gill, 2004). Microfiltered WPIs (100 μg/mL) have been reported to significantly stimulate the proliferation of lymphocytes during in vitro studies (Mercier et al., 2004). On supplementation of immunostimulatory WPC (10.5 g/100 g of diet for 4 weeks) in milk powder‐based diets of mice, there was significant enhancement in humoral immune response along with improvement in antibody responses to orally administered antigens (Rutherfurd‐Markwick et al., 2005). On the other hand, ex vivo analysis of this study indicated enhancement of splenic lymphocytic proliferation along with phagocytic activity of leukocytes in blood and peritoneal cavity. According to an in vitro study, it was found that bovine LF (BLF) and bovine LPO (BLPO) fractions of whey had a substantial boosting effect on the formation of neutrophil superoxides, indicating that these milk protein fractions indeed contribute to the immunoenhancing features of whey (Wong, Liu, et al., 1997). Bounous et al. (1993) reported that feeding mice with an undenatured WPC for 4 weeks resulted in production of higher number of helper T cells and a higher proportion of helper to suppressor cells in mice than those fed with a diet‐containing isocaloric CN. Thus, isolating and characterizing individual bioactive peptides from WPs is necessary for studying their immunobiological properties, and exploiting their use in viral affections including COVID‐19.

According to some reports, WPs, that is, α‐lactalbumin (ALA), β‐lactoglobulin (BLG), and bovine gamma globulin (BGG), act as immunostimulatory in murine spleen cells, and purified proteins dramatically increase IgM synthesis and cell proliferation (Wong et al., 1998). Two lactoimmunopeptides, that is, “Tyr‐Gly (f50‐51, f18‐19)” and “Tyr‐Gly‐Gly (f18‐20),” derived from the N‐terminal of ALA have been reported to stimulate the proliferation and protein synthesis of HPBL activated with concanavalin A in cell culture (Kayser & Meisel, 1996). BLG, in undenatured form, is the main and primary milk ingredient that improves immunological responses by modulating cell proliferation through the IgM receptors (Tai et al., 2016). The immunostimulatory role of BLG is also well documented, and it triggers cellular activation in immune cells from both humans and mice (Bounous et al., 1989; Tai et al., 2016). Brix et al. (2003) observed that cells from spleen and mesenteric lymph nodes proliferated noticeably when exposed to commercial preparations of BLG. In addition, these proteins elevated the intracellular GSH concentration in splenic cell cultures.

Lactoferrin is another valuable AM protein that specifically binds to human neutrophils and B lymphocytes (Iyer & Lonnerdal, 1993). However, there is an indication of presence of receptors for BLF on the plasma membranes of polymorphonuclear cells also (Maneva et al., 1994). Thus, BLF and/or its fractions may have immunoregulatory roles both at the levels of blood and intestinal mucosa. In general, administration of LF may protect from respiratory viral infections by boosting the systemic immune response (enhancing NK cell activity and Th1 cytokine response) and preventing viral attachment and replication inside the host cells (Wakabayashi et al., 2014). Both human LF and BLF may affect cells of the adaptive immune system in addition to increasing the number of cytotoxic cells necessary for the innate immune system, such as NK cells (Actor et al., 2009). LF promotes the differentiation of immature B cells into effective antigen‐presenting cells (APCs) and stimulates the development of T cell precursors into competent helper cells (Sienkiewicz et al., 2022). Oral supplementation of BLF (up to 200 mg) has resulted in significant enhancement in the count of cytotoxic, helper, and total T cells (Mulder et al., 2008). Another study confirmed the suppression of cold‐associated symptoms (common cold, cough, sore throat, nasal congestion, watery eyes, sputum, headache, and fatigue) and gastrointestinal symptoms (anorexia, diarrhea, and stomach pain) by oral administration of BLF at the dose rate of 100–1000 mg/day/individual (Egashira et al., 2007; Oda et al., 2012; Vitetta et al., 2013). Since these types of symptoms are commonly observed in COVID‐19 (Czubak et al., 2021), hence LF, which is inexpensive, easily available and generally recognized as safe (GRAS) molecule, can be used as a nutritional adjunct for COVID‐19.

The above findings indicate that milk proteins may have a potential to promote immunological stimulation, and thus they could be used in various forms for prophylactic and therapeutic management of COVID‐19.

#### 2.2.2 Antiviral properties

The antiviral roles of ALA and BLG (Table 3), mucins and glycoprotein fractions (Table 4), LF (Table 5), and LPO (Table 6) against some important pathogenic viral diseases of humans are well known. All these WPs may also act as antidotes to SARS‐CoV‐2 (Gallo et al., 2022). Because of their strong antiviral characteristics against SARS‐CoV‐2, WPs and peptides piqued researchers' curiosity the most. Human CoVs including SARS‐CoV‐2 cause infection by binding to various host cell receptors, that is, furin, angiotensin‐converting enzyme 2 (ACE2), and DPP‐4, and employ them to enter target cells (Johnson et al., 2020; Noh et al., 2021). Thus, corresponding receptor inhibitors will not allow cleavage of S1/S2 domain of spike protein subunit of SARS‐CoV‐2, and block the entry of virus into the host cells (Cheng et al., 2020). Moreover, for the treatment of CoV infections, viral 3C^pro^ (3C protease) or 3CL^pro^ (3‐chymotrypsin‐like cysteine protease, also known as main protease, i.e., M^pro^) is the most thoroughly studied therapeutic drug target because of its critical function in processing of viral polyproteins into mature proteins (Cannalire et al., 2020; Tan et al., 2023). During the COVID‐19 pandemic, several mutations were noticed in the binding regions of the spike protein receptors (SPRs) and M^pro^ genes of different SARS‐CoV‐2 variants (Hu et al., 2022; Jukic et al., 2021; Sacco et al., 2022). Although M^pro^ and PL^pro^ inhibitors may serve as effective tools against different SARS‐CoV‐2 variants, emerging mutations raise serious concerns about their potential therapeutic resistance. The potential advantage of exploring AM as antivirals is that it might be active against multiple variants.

**TABLE 3:** Antiviral roles of whey proteins (α‐lactalbumin and β‐lactoglobulin) against some important pathogenic viruses of humans.

S. No	Type of whey proteins	Name of virus	In vitro mechanism of action	References
1.	Bovine ALA and BLG (methylated)	PV‐1 and Coxsackie virus B6	Inhibition of virus entry, replication, transcription, and translation	Sitohy et al. (2008)
2.	Bovine ALA and BLG (methylated)	HCV	Interaction with viral genome and disrupting the virus's transcription or replication processes.	Chobert et al. (2007)
3.	ALA (methylated) and BLG (methylated and ethylated)	HSV‐1	Blocking the virus entry into host cells and preventing interaction between the viral and cellular proteins	Sitohy et al. (2007)
4.	ALA and BLG (chemically modified)	HSV‐1	Inhibiting virus multiplication	Oevermann et al. (2003)
5.	ALA and BLG (chemically modified)	Avian influenza A (H5N1)	Preventing interaction with viral nuclear proteins (PB1, PA, NP, PB2, PA, and NP) and disrupting the entire replication cycle	Taha et al. (2010)
6.	Bovine BLG (methylated)	Influenza virus A (H1N1)	Suppressing viral RNA replication	Sitohy et al. (2010)
7.	Bovine BLG	Human rotavirus	Inhibiting hemagglutination as well as virus binding to host cell receptors	Superti et al. (1997)
8.	Bovine BLG (3‐hydroxyphthaloyl)	HSV‐1, HSV‐2	Binding to virus particles	Neurath et al. (1998)
9.	Bovine BLG (chemically modified)	HPV‐6, HPV‐16, and HPV‐18	Inhibiting early stage of virus replication mainly entry process of virus	Lu et al. (2013)

Abbreviations: ALA, α‐lactalbumin; BLG, β‐lactoglobulin; HCV, human cytomegalovirus; HPV, human papillomavirus; HSV, herpes simplex virus; PV, poliovirus; RNA, ribonucleic acid.

**TABLE 4:** Antiviral roles of milk mucins and other glycoprotein fractions against some important pathogenic viruses of humans.

S No.	Type of mucins/glycoproteins	Name of virus	In vitro mechanism of action	References
1.	High‐M_r_ mucin‐like glycoprotein fraction (cow milk)	Human rotavirus	Inhibiting virus replication step	Kanamaru et al. (1999)
2.	bMUC1, CM3Q3 (MMWP)	Human rotavirus	Inhibition of virus infection particularly in intestinal cell lines	Bojsen et al. (2007)
3.	bMUC1/MMWP	Human rotavirus	Intervention with infection process before as well as after virus–host cell attachment	Kvistgaard et al. (2004)
4.	b/oMUC1	Rotavirus	Inhibition of viral infection	Parron et al. (2016)
5.	b/oLad	Rotavirus	Inhibition of viral infection	Parron et al. (2016)
6.	bLad	Human rotavirus	Inhibition of viral infection	Inagaki et al. (2010)
7.	eLad‐derived peptides	Human rotavirus	Inhibiting attachment of virus‐to‐cell surface	Civra et al. (2015)

Abbreviations: b/oLad, bovine/ovine lactoadherin; b/oMUC1, bovine/ovine mucin 1; bLad, bovine lactadherin; bMUC1, bovine mucin 1; eLad, equine lactoadherin; MMWP, macromolecular whey proteins.

**TABLE 5:** Antiviral roles of milk lactoferrin against some important pathogenic viruses of humans.

Lactoferrin	Name of virus	In vitro mechanism of action	References
BLF	SARS‐CoV	Blocking the primary interaction between SARS‐CoV and host cells by binding to HSPGs in addition to enhancement of natural killer cell activity and stimulation of neutrophil aggregation	Lang et al. (2011)
	HCoV‐229E, HCoV‐NL63, HCoV‐OC43, SARS‐CoV‐2	Inhibiting virus attachment to host cell by binding with HSPGs	de Carvalho et al. (2020); Hu et al. (2021); Salaris et al. (2021); Wotring et al. (2022)
	Dengue virus	Interacting with heparan sulfate, low‐density lipoprotein receptors, and DC‐SIGN (dendritic cell‐specific intercellular adhesion molecule‐3‐grabbing non‐integrin)	Chen et al. (2017)
	Influenza A virus	Blocking nuclear export of viral ribonucleoproteins and preventing viral assembly, and inhibition of virus‐induced apoptosis	Pietrantoni et al. (2010)
	Influenza A (H1N1 and H3N2) viruses	Binding to the HA(2) region of viral hemagglutinin and suppressing virus‐induced hemagglutination and infection	Ammendolia et al. (2012)
	Parainfluenza virus 2	Inhibition of entry of virus into the cells by binding to cell surface along with partial inhibition of viral RNA and protein synthesis	Yamamoto et al. (2010)
	Hepatitis B virus	Mechanically block the normal process of viral adhesion	Hara et al. (2002)
	Hepatitis C virus	Binding with envelope proteins (E1 and E2) of viral particles, neutralizing the virions, and preventing their adsorption into cultured human hepatocytes	Tanaka et al. (1999)
	Japanese encephalitis virus	Inhibition of infection by binding to cell surface expressed glycosaminoglycans (heparan sulfate) and receptors for low‐density lipoprotein	Chien et al. (2008)
	Chikungunya and zika viruses	Antiviral role at two steps—binding/entry (due to blockage of heparin sulfate) and production/exit of virus (due to RNase activity and degradation of virus RNA)	Carvalho et al. (2017)
	Echovirus 6	Interaction with viral structural polypeptides and inhibition of endocytic pathway	Ammendolia et al. (2007)
	Hantavirus (SR‐11)	Inhibiting invasion in host cells and viral shedding	Murphy et al. (2000)
	Adenovirus	Binding to viral particles in addition to targeting viral III and IIIa structural polypeptides during replication	Pietrantoni et al. (2003)
	HIV‐1	Blocking viral entry into host cells and CXCR4 or CCR5 attachment and suppressing the further multiplication	Berkhout et al. (2002)
	Enterovirus 71 and Coxsackievirus A16, PV‐1	Inhibition of viral adsorption into host cells	Lin et al. (2002); Marchetti et al. (1999)
	HSV‐1, HSV‐2	Preventing virus attachment by binding to host cell surface and targeting viral adsorption	Marchetti et al. (2009)
	Toscana virus	Inhibiting the viral adsorption step by competitively binding to heparan sulfate	Pietrantoni et al. (2015)
	Human norovirus	Interfering with the uncoating process of virus and hampering viral replication	Oda et al. (2021)
OLF	Hepatitis C virus genotype 4a	Preventing replication by blocking the entry to HepG2 cells	El‐Fakharany et al. (2013)
CPLf	HPV	Preventing viral entry by directly binding to them	Yugis et al. (2015)
CMLf	Hepatitis C virus genotype 4	Inhibition of entry of viral particles into human peripheral blood mononuclear cells (PBMC), HepG2 cells, and suppression of replication	Redwan and Tabll (2007)

Abbreviations: BLF, bovine lactoferrin; CMLF, camel lactoferrin; CPLF, caprine lactoferrin; HCoV, human coronavirus; HIV, human immunodeficiency virus; HPV, human papillomavirus; HSPG, heparan sulfate proteoglycans; HSV, herpes simplex virus; OLF, ovine lactoferrin; PV, poliovirus; RNA, ribonucleic acid; SARS‐CoV, severe acute respiratory syndrome corona virus.

**TABLE 6:** Antiviral roles of milk lactoperoxidase against some important pathogenic viruses of humans.

S No.	Type of lactoperoxidase	Name of virus	In vitro mechanism of action	References
1.	BLPO	PV‐1 and vaccinia virus	Decreasing infectiousness and cytopathic effect	Belding et al. (1970)
2.	BLPO	HIV‐1	Inhibiting viral replication and cytopathic effects on CEM and HUT 78 cell lines	Yamaguchi et al. (1993)
3.	BLPO	Human influenza virus A and B	Attachment of hypothiocyanite (OSCN^−^) ions with virus envelope and dramatically reducing the formation of plaques in MDCK (Madin–Darby canine kidney) cell lines	Sugita et al. (2018)
4.	HLPO, BLPO, CMLPO	HSV‐1	Inhibiting the growth of virus on vero cells	El‐Fakharany et al. (2017)
5.	HLPO, BLPO, CMLPO	Hepatitis C virus genotype 4	Preventing viral replication or neutralization in HepG2 cells by blocking the virus receptors on the cell surface	Redwan et al. (2015)
6.	BLPO	Influenza virus	Virucidal activity against virus	Shin et al. (2005)
7.	BLPO	HSV‐1, RSV, and Echovirus type 11	Virucidal activity of hypothiocyanous acid/hypothiocyanite ions in human gingival fibroblast cells	Mikola et al. (1995)
8.	BLPO	Adenovirus and RSV	Preventing the release of virus from cells by hypoiodous acid and inhibition of synthesis or assembly of viral nucleic acids and proteins	Fischer et al. (2011)
9.	BLPO	Vaccinia virus	Inhibiting growth of virus particles	Tanaka et al. (2012)

Abbreviations: BLPO, bovine lactoperoxidase; CMLPO, camel lactoperoxidase; HIV, human immunodeficiency virus; HLPO, human lactoperoxidase; HSV, herpes simplex virus; PV, poliovirus; RSV, respiratory syncytial virus.

Whey proteins (LF, ALA, and mucin1) obtained from human breast milk showed strong antiviral roles against different SARS‐CoV‐2 variants (α, β, γ, and κ), as these can inhibit viral infection at all stages of replication cycle, that is, attachment [by binding to heparan sulfate proteoglycans (HSPG) receptors], entrance, and postentrance replication (Lai et al., 2021). Two peptides, that is, “Ala‐Leu‐Pro‐Met‐His‐Ile‐Arg” and “Ile‐Pro‐Ala‐Val‐Phe‐Lys,” isolated from BLG of goat milk, and were reported to block SARS‐CoV‐2 entrance into host cells by showing inhibitory effects on ACE2 and DPP‐4 receptors, as analyzed on the basis of docking scores in an in silico study (Cakir et al., 2021). Gambacorta et al. (2022) studied the anti‐SARS‐CoV‐2 potential of BLG‐derived three peptides, which also possess strong ACE inhibitory properties. The molecular study proved certain interactions between these peptides and amino acid sequences of 3CL^pro^ in terms of docking scores and binding free energy values. In silico studies have also documented the ability of these peptides to inhibit interaction between spike proteins of SARS‐CoV‐2 and DPP‐4 receptors; however, these observations are yet to be confirmed in in vitro and in vivo studies. Behzadipour et al. (2021) also documented the antiviral role of milk peptides obtained by the proteolysis of bovine milk CNs and WPs. As per molecular docking analysis, a total of five peptides, that is, P1, P18 (BLG), P3 (β‐CN), P17 (α_S1_‐CN), and P20 (α_S2_‐CN), exhibited SARS‐CoV‐2 Mpro inhibitory activity in terms of strongest degree of interaction and binding affinity with the residues in its active‐site cleft. Whey peptides (4–13 residues) isolated from buffalo milk and colostrum have also been reported to show antiviral activity against SARS‐CoV‐2 in various ways, that is, inhibiting replication transcription complex, virion assembly, and endosomal maturation in addition to blocking viral entrance by binding to spike S and ACE2 (Pradeep et al., 2021). Recently Tufan et al. (2022) reported that administration of WPC at the dose rate of 2 g/kg for 10 days reduced the oxidative lung injury caused by methotrexate and prevented lung furin activity and the binding of SARS‐CoV‐2 spike protein to ACE2 receptors. Different whey‐derived peptides, that is, “IPP,” “IIAE,” “LIVTQ,” and “LVYPFP,” may inhibit SARS‐CoV‐2 entrance by suppressing ACE2 through precise molecular interactions with important ACE2 residues (Chamata et al., 2021). However, further in vitro and in vivo research is required to confirm the underlying mechanisms through which these peptides block ACE2.

**FIGURE 1:**
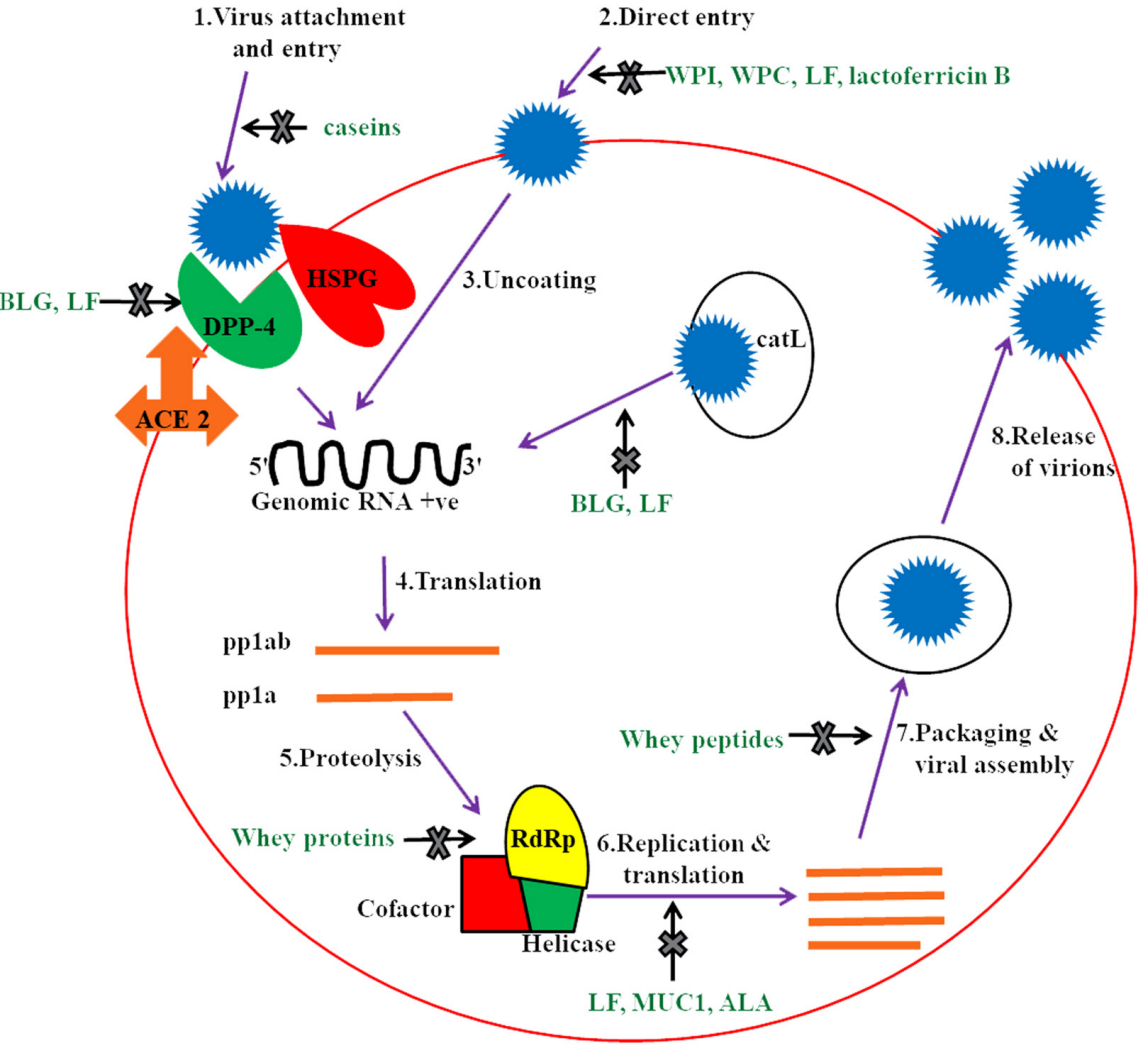
Mechanism of actions of different milk proteins and peptides acting at various stages of SARS‐CoV‐2 life cycle.

Since 1987, the antiviral activity of milk LF is well‐known due to its inherent ability to bind to both HSPG receptors of host cells and/or surface elements of viral particles. LF shows antiviral activity against a number of DNA and RNA viruses, including herpes simplex virus (HSV), respiratory syncytial virus (RSV), human immunodeficiency virus (HIV), rotavirus (Table 5), and SARS‐CoV‐2 (Elnagdy & AlKhazindar, 2020). This mysterious AM protein acts mainly in the initial stage of viral infections either by direct attachment to the virus particles or by preventing the virus internalization via blockage of host cell receptors (Van der Strate et al., 2001). Bovine milk LF (50% inhibitory concentration of 0.7 mM) was reported to inhibit in vitro cell entry of SARS pseudovirus (closely related to SARS‐CoV‐2) by binding to HSPG receptors (Lang et al., 2011). However, molecular modeling suggests that BLF can inhibit SARS‐CoV‐2 either by directly binding to viral particles via their spike S glycoproteins or by suppressing the binding of spike protein to the ACE2 receptors (Campione et al., 2021; Hu et al., 2021). Overall, LF can act even in the nanomolar range in different cell models with various mechanisms of action, including preventing viral infection and augmenting interferon responses. Fan et al. (2020) reported the in vitro antiviral effect of WPs against two similar CoVs, that is, SARS‐CoV‐2 and pangolin CoV, as both share approximately 92.2% amino acid similarity in their spike proteins. They demonstrated that human breast milk showed highest antiviral efficacy against SARS‐CoV‐2 and pangolin CoV than cow and goat milk. The study also proved that individually recombinant, BLF and human LF (at the concentration of 1 mg/mL) partially inhibited these viral infections, indicating the presence of some other antiviral ingredients in breast milk except LF. These milk proteins variably inhibited attachment, RNA‐dependent RNA polymerase (RdRp) activity, and postentry replication stages of CoVs. WPI, WPC, BLF, and lactoferricin B (a proteolytic peptide generated from BLF) displayed in vitro anti‐SARS‐CoV‐2 activity (by inhibiting direct entry of virus) against different strains of CoVs originating from India, Brazil, United Kingdom, and South Africa (Wotring et al., 2022). The antiviral activity of BLF against human CoVs (HCoV‐OC43, HCoV‐229E, HCoV‐NL63, and SARS‐CoV‐2) was found to be higher than human LF as demonstrated in cell culture studies (Hu et al., 2021). Some of the investigations documented that BLF not only exhibited synergism with remdesivir but also enhanced its effectiveness by around eight times in cell culture techniques (Hu et al., 2021; Mirabelli et al., 2021). According to an in vitro hypothesis, both LF and BLG have ability to inhibit cathepsin L, thereby suppressing the proteolysis process in viral infection, and thus decreasing the virus internalization (Madadlou, 2020). Hence, due to strong antiviral properties of LF, it may be used as an immunity enhancer or as a drug in combination with traditional antivirals or both. Moreover, it will be preferable to all other management techniques due to its accessibility, environmental safety, and effectiveness. Figure 1 demonstrates mechanism of actions of different milk proteins and peptides acting at various stages of SARS‐CoV‐2 life cycle.

Although the studies showing the antiviral potential of AM‐originated lysozyme (LYS) and LPO against SARS‐CoV‐2 are lacking, the efficacy of these two molecules (nonmilk origin) has been proved well at entry points (mouth, conjunctiva, and nasal cavity) of SARS‐CoV‐2 virus. According to a speculative theory, LYS found in tears, has power to prevent SARS‐CoV‐2 from entering the cornea and conjunctiva (de Freitas Santoro et al., 2021). Similarly, LYS pretreatment in human corneal epithelial cells reduced the entry of SARS‐CoV‐2 into normal (in a dose‐dependent manner) as well as inflammatory cells (Song et al., 2022). The enzyme LPO catalyzes the formation of hypothiocyanite (OSCNˉ) ions from the oxidation of thiocyanate in the presence of H_2_O_2_. The antiviral action of OSCN is attributed to interference with synthesis and assembly of viral proteins and nucleic acids (Cegolon & Mastrangelo, 2020). An in vitro investigation demonstrated the time‐ and dose‐dependent viral killing action of OSCNˉ against SARS‐CoV‐2, which was marginally increased by the concurrent existence of Lf (Cegolon et al., 2021).

#### 2.2.3 Anti‐inflammatory properties

The mortality in COVID‐19 is associated with “cytokine storm syndrome (CSS),” which is characterized by the robust and uncontrolled secretion of cytokines, tumor‐necrosis factors, interleukins, interferons, chemokines, and several other mediators as a result of exaggerated immune response (Sinha et al., 2020). If uncontrolled, this hyperinflammatory phase results in death of patient due to development of acute respiratory distress syndrome (ARDS) and multiorgan failure (Ragab et al., 2020). Thus, control of this phase by using immunosuppressants and anti‐inflammatory agents is of utmost importance in the later stages of disease.

Milk protein LF acts like a chameleon, as it can act both as an immunostimulant as well as immunosuppressant (Krissansen, 2007). A number of in vitro and in vivo studies documented that LF may suppress the release of proinflammatory cytokines (IL‐1, IL‐6, and TNF‐α), as well as enhance the production of anti‐inflammatory molecules (IL‐10) (Berlutti et al., 2006; Chea et al., 2018; Haversen et al., 2002; Hu et al., 2020; Kruzel et al., 2002; Valenti et al., 2017). A number of complex mechanisms are responsible for this activity of LF, namely reducing the generation of ROS due to its iron scavenging properties (Siqueiros‐Cendon et al., 2014), inhibiting the sepsis development by preventing the formation of CD14‐LPS complex and activation of Toll‐like receptor‐4 signaling pathways (Yen et al., 2011), controlling the activity of cell signaling pathways via regulation of cell surface receptors and maintenance of general physiological homeostasis in the body (Actor et al., 2009), and capacity to alter the proportion of Th1 and Th2 cells subsets, which limits uncontrollably high inflammatory responses (Fischer et al., 2006). It is noteworthy that the immunological health of the host may play a major role in this range of LF actions. On treatment of mitogen‐activated T cells with BLF, there was reduction in total cytokine secretion due to suppression of intracellular signaling that occurred as a result of interaction between the mitogen and its receptor (Kobayashi et al., 2005). According to an in vivo study, BLF significantly reduced the secretion of proinflammatory cytokines (IL‐6 and TNF‐α) from the splenocytes of jaundiced rats (Zimecki et al., 2003). The above observations indicate that LF may control CSS stage of severe COVID‐19, and may act as a clinical candidate for treatment purposes.

Lysozyme, which is present in significant amounts in breast milk, has been reported to inhibit neutrophil chemotaxis as well as production of hazardous oxygen molecules during phagocytosis (Goldman et al., 1990). Other anti‐inflammatory properties of LYS include suppression of mitogen‐induced lymphoblastogenesis and the autologous mixed lymphocyte reaction (at doses ranging from 1 to 10 mg/mL) (Ogundele, 1998). Recombinant human LYS is an intriguing possibility for the treatment of pulmonary infections including pneumonia (Griswold et al., 2014). According to a study, pretreatment of human corneal epithelial cell lines with LYS (10 mg/mL) resulted in significant reduction in the levels of proinflammatory cytokines, that is, IL‐6, IL‐8, IL‐1β, TNF‐α, and MCP‐1 (monocyte chemo attractant protein‐1), induced by spike proteins of SARS‐CoV‐2 (Song et al., 2022). These functions of LYS may make it a suitable candidate for management of hyperinflammatory stage during COVID‐19, but it needs further investigation.

Lactoperoxidase, the second‐most prevalent whey enzyme in bovine milk, possesses indispensable anti‐inflammatory properties. LPO causes removal of free radicals, that is, H_2_O_2_ and NO from the inflammation site by catalyzing their conversion into antimicrobial hypothiocyanate anions using thiocyanate (Kussendrager & van Hooijdonk, 2000). Oral administration of BLPO in mice (62.5 mg/body/d) having dextran sulfate sodium‐induced colitis resulted in significant suppression of inflammation, proinflammatory cytokine (IL‐6), and intestinal crypt damage scores (Shin et al., 2008). This may be of significant use in therapeutic management of COVID‐19, as SARS‐CoV‐2 may trigger ulcerative colitis in patients manifesting gastrointestinal symptoms (Aydin & Taşdemir, 2021; Mazza et al., 2020; Rosen et al., 2020). Shin et al. (2005) found that oral administration of BLF and BLPO decreased the infiltration of inflammatory cells, suppressed pneumonia, and drastically decreased lung consolidation scores in BALB/c mice infected with the influenza virus strain. In addition, BLPO significantly reduced the level of serum proinflammatory cytokine (IL‐6) in mice than control on Day 6. Another study found that oral consumption of LF and LPO by persons who did not regularly gargle or wear a face mask experienced symptoms of the common cold and fever less frequently and for a shorter period of time (Shin et al., 2018). All these results of clinical studies clearly indicated that LPO may also yield beneficial results in terms of anti‐inflammatory agents during clinical management of COVID‐19.

## 3 MILK CARBOHYDRATES

### 3.1 Milk oligosaccharides (Milk glycans)

Milk oligosaccharides (MOs) possess several biological activities including selective enrichment of beneficial bacteria, inhibiting binding of undesirable or pathogenic bacteria to colonocytes, anti‐inflammatory properties, glycome‐modifying activity, development of brain and immune system, and growth‐related characteristics of intestinal cells (Oliveira et al., 2015). The qualitative and quantitative composition of oligosaccharides in AM is variable due to many reasons, namely type of analytical techniques, oligosaccharide extraction methods, genetic variations in animals, and stage of lactation (Barile et al., 2010; Tao et al., 2009). The concentrations of oligosaccharides in bovine, equine, caprine, ovine, and camel milks are 0.03–0.06, 0.0798–0.2178, 0.25–0.30, 0.02–0.04, and 1.2 g/L, respectively (Karav et al., 2018; Oliveira et al., 2015). According to different studies, approximately >200 human MOs (HMOs) (Albrecht et al., 2014), 40 bovine MOs (BMOs) (Tao et al., 2008), 43 equine MOs (EMOs) (Difilippo et al., 2015), 20 caprine MOs (CpMOs) (Martinez‐Ferez et al., 2006), 29 porcine MOs (PMOs) (Tao et al., 2010), and 12 camel MOs (CmMOs) (Alhaj et al., 2013) have been identified in milk obtained from human, bovine, caprine, porcine, and camel, respectively. However, out of these nearly 10 BMOs and 9 CpMOs have structures that are similar to some HMOs, suggesting that they may have shared functionalities (Quinn, 2021).

#### 3.1.1 Antiviral properties

There are a number of studies that document the promising antiviral nature of HMOs against many pathogenic viruses such as RSV and influenza virus (Duska‐McEwen et al., 2014), rotavirus (Yu et al., 2014), norovirus (Koromyslova et al., 2017), and HIV‐1 (Hong et al., 2008) in humans. Nguyen et al. (2021) observed acidic HMOs at high relative abundances in CaR‐ESI‐MS screening, thereby raising the possibility that neonates may be protected against SARS‐CoV‐2 by breastfeeding. Moreover, according to an observational cohort study, it was confirmed that 72 neonates, who were receiving breastfeeding from SARS‐CoV‐2‐positive mothers, were all tested negative after 14 days of life (Salvatore et al., 2020).

In milk of all domestic animals, about 80%–90% of the total oligosaccharides are sialylated, containing N‐acetylneuraminic acid (Neu5Ac) and/or N‐glycolylneuraminic acid (Neu5Gc) (Albrecht et al., 2014). Humans lack ability to synthesize Neu5Gc, which is appreciably produced in bovine milk (Padler‐Karavani & Varki, 2011), and this structure is suggested to play a role in chronic inflammation‐mediated diseases (Okerblom & Varki, 2017). Sialic acid residue, that is, Neu5Ac, is significantly more abundant than Neu5Gc in BMOs (Tao et al., 2008). Being highly sialylated, BMOs mainly target sialic acid‐dependent pathogens. HCoVs generally rely on assistance of glycans for cell entry, for example, MERS‐CoV binds sialylated glycans to facilitate host cell entry (Li, Hulswit, et al., 2017), HCoV‐OC43 and HKU1 engage sialoglycans with 9‐*O*‐acetylated sialic acid as key receptors (Tortorici et al., 2019), SARS‐CoV‐1 and CoV‐NL63 exploit acidic heparan sulfate (HS) polysaccharides (Milewska et al., 2014), and SARS‐CoV‐2 also binds and enters host cells via glycans like heparan sulfate (HS) and sialic acid‐containing glycolipids/glycoproteins (Hao et al., 2021). According to Errasfa (2021), MOs from camel's and donkey's milk, may bind to virus lectin glycoproteins in addition to their prebiotic role. Thus, MOs may exert an ideal decoy role against SARS‐CoV‐2 entry into the cells. Therefore, CmMOs and donkey's MOs may act as genuine tools against SARS‐CoV‐2, but there is further need for investigation in clinical trials as purified components or as part of treatment using whole AM from these animals. From the above discussion and observations, it could be speculated that HMOs and animal MOs may act as antiviral molecules and offer some protection against SARS‐CoV‐2, but it needs further research in this direction before arriving at final conclusion.

### 3.2 Glycosaminoglycans

Glycosaminoglycans (GAGs), also known as mucopolysaccharides, are negatively charged, sulfated linear polysaccharide molecules such as heparin/heparan sulfate (HP/HS), hyaluronic acid (HA), chondroitin sulfate (CS), and dermatan sulfate (DS) present in AM. Compared to bovine milk, human milk contains more concentration of GAGs (approximately seven times), with DS making up 40% of all whole GAGs in bovine milk and HS/HP, CS, and other GAGs making up the remaining 30% and 21%, respectively (Coppa et al., 2013). The higher concentration of GAGs in human milk indicates their importance in first 2 weeks of infant life. Milk GAGs are produced inside mammary glands with a specific core protein linked with their long chains.

#### 3.2.1 Antiviral properties

In context with COVID‐19 disease, milk GAGs may serve two important functions. Firstly, during digestion in small intestine, their associated core protein gets digested by proteolytic enzymes and remaining GAG chains reach the large intestine in intact form. Thus, microorganisms in the intestinal tract utilize these GAGs as prebiotics to facilitate growth (Newburg & He, 2015). This maintains healthy gut by preventing various enteric bacterial and viral infections. Secondly, although antiviral activities of commercially available GAGs have been reported for some of the viruses, for example, commercial CS for dengue virus (Kato et al., 2010), human milk CS for HIV‐1 (Newburg et al., 1995), and commercial HP for HIV‐1 (Baba et al., 1988), HSV (Laquerre et al., 1998), Zika virus (Ghezzi et al., 2017), and SARS‐CoV (Vicenzi et al., 2004). The antiviral potential of GAGs present in bovine milk needs to be fully characterized. Moreover, the presence of similar carbohydrate structures between different GAGs may explain their viral inhibitory activity against multiple viruses. Free HS has been reported to inhibit SARS‐CoV‐2 infection of Vero cells (Kwon et al., 2020). Also, HS increases the affinity of SARS‐CoV‐2 RBD for ACE2, indicative of HS acting as a more classical coreceptor (Clausen et al., 2020). According to Vicenzi et al. (2004), SARS‐associated CoV infection was inhibited by 50% upon incubation of Vero cells with heparin (100 μg/mL) 30 min before viral addition. This partial inhibition of SARS‐CoV infection might be due to the interaction between positively charged amino acids (present on virus envelope) with negatively charged sulfate groups present on HS proteoglycans expressed on the surface of target cells. Thus, GAGs may show antiviral action against members of Coronaviridae including SARS‐CoV‐2, the causative agent of COVID‐19 pandemic. Further exploration and investigation regarding the potential of GAGs to block this deadly virus could be interesting in future research.

## 4 MILK FATS

### 4.1 Glycerol monolaurate

Glycerol monolaurate (GML) (2,3‐dihydroxypropyl dodecanoate) is a fatty acid monoester that possesses broad antimicrobial (against bacteria, fungi, and enveloped viruses), anti‐inflammatory, and immunoregulatory properties (Schlievert et al., 1992, 2019; Witcher et al., 1996). This molecule has been accorded GRAS status and is commonly used in cosmetics, as a homeopathic supplement, food preservative, and emulsifier (Zhang et al., 2016). The concentrations of GML in bovine milk, whole pasteurized human milk, and infant formula are estimated as 0.15, 3, and 0 mg/mL, respectively (Schlievert et al., 2019).

#### 4.1.1 Immunomodulator properties

As an immunostimulant, GML enhances the immune system by modulating immune system reactions, activating and attracting leukocytes to the site of infection along with limited ROS production, thereby causing less tissue damage (Subroto & Indiarto, 2020). Human primary T cells are impacted by GML in terms of signaling and functional output (Zhang et al., 2016). In addition, it has been reported to reduce T cell receptor‐induced production of proinflammatory cytokines including IL‐1 α, IL‐1β, IL‐2, IL‐6, IL‐8, IL‐10, MIP‐3α (macrophage inflammatory protein‐3α), TNF‐ α, and IFN‐γ (Li et al., 2009; Witcher et al., 1996). Thus, GML may also play potential role during CSS stage of COVID‐19.

#### 4.1.2 Antiviral properties

Glycerol monolaurate has been shown to possess antiviral activity against a number of enveloped viruses, such as HIV‐1, HSV‐1, measles, cytomegalovirus, yellow fever virus, mumps virus, Zika virus, influenza, and CoVs (Subroto & Indiarto, 2020). However, it did not show any inhibitory effect on nonenveloped viruses indicating its association with direct viral envelope interference and modulatory changes in it, thereby preventing the binding of the virus to the host cell membrane, or inhibiting the RNA synthesis and viral maturation (Welch et al., 2020). Electron microscopies of Hierholzer and Kabara (1982) revealed loss of virus infectivity due to generalized disintegration of envelope when influenza A and CoV were coincubated with a GML mixture on primary rhesus monkey kidney (MK) cells, a human laryngeal epidermoid carcinoma cell line (HEp‐2), and a human embryonic lung diploid fibroblast cell strain (HELF). SARS‐CoV‐2 also possesses characteristics similar to that of enveloped viruses including membrane and core of virus composed of phospholipids and RNA genome, respectively. Additionally, SARS‐CoV‐2 principally targets the respiratory system, and its characteristics are closely related to the SARS virus which caused pandemic in 2003 (Kang et al., 2020; Zhou et al., 2020). Thus, SARS‐CoV‐2, being an enveloped virus, may also act as target for GML. The above discussion provides evidence for further investigation into this class of compounds for the potential treatment of COVID‐19.

### 4.2 Polyunsaturated fatty acids

Among long‐chain polyunsaturated fatty acids (LC‐PUFAs), DHA (docosahexaenoic acid), and EPA (eicosapentaenoic acid) are predominant ones. Milk and milk products are considered not to contribute significantly to dietary intake of ω‐3 fatty acids (Van Valenberg et al., 2013). In general, milk from cows fed normal diets (forages and/or cereals) contains extremely low concentrations of EPA and DHA (less than 1 g/100 g of fatty acids) (Givens & Shingfield, 2006). However, the concentration of both DHA and EPA can be increased in milk by including some fish oil in the diet of the cow, with a typical efficiency of transfer of EPA and DHA from the diet into milk as 2.6% and 4.1%, respectively (Chilliard et al., 2000). Cattaneo et al. (2006) fed fish oil (1.1% of total mixed ration) to dairy goats and reported increase in DHA and EPA contents of milk from 0.07% (control) to 0.51% and 0 (control) to 0.50% of total fatty acids, respectively. Similarly, feeding fish meals to dairy cows resulted in increase in DHA content of milk from 0.26 to 0.72% of total fatty acids (Wright et al., 2003). Hence, enrichment of AM and milk products is mandatory for getting their optimum health benefits.

#### 4.2.1 Immunomodulator properties

Evidence suggests that among ω‐3 LC‐PUFAs, both DHA and EPA have direct influence on the immunological response to viral infections (Calder et al., 2020; Messina et al., 2020). Cells of immune system contain a high content of EPA and DHA in their membranes (Miles et al., 2021). In blood mononuclear cells (a mixture of lymphocytes and monocytes) of adults, DHA and EPA typically comprise 2%–3% and 0.5%–1%, respectively (Rees et al., 2006; Yaqoob et al., 2000). Within the immune cell membranes, these LC‐PUFAs form signaling platforms known as lipid rafts and modulate intracellular signaling, ultimately affecting transcription factor activation and gene expression (Calder, 2015). As a result of these effects, LC‐PUFAs regulate the function of various immune cell types including neutrophils, monocytes, macrophages, dendritic cells, T cells, and B cells (Calder, 1998). As a result, ω‐3 fatty acids aid in the reduction of inflammation, which in turn supports a healthy immune system. For this, 250 mg/day of EPA and DHA must be consumed every day, which can be easily provided by inclusion of enriched AM and milk products in the diet (Calder et al., 2020).

#### 4.2.2 Anti‐inflammatory properties

A number of proinflammatory mediators can produce inflammation, which is a crucial part of the immune response. However, due to the activation of particular negative feedback systems, it ends fast toward the conclusion of the immunological response. The anti‐inflammatory effects of EPA and DHA are mediated by intricate cascades of events including inhibition of leukocyte chemotaxis, reduction in adhesion molecule expression and leukocyte–endothelial adhesive interactions, disruption of lipid rafts, inhibition of activation of NF‐κB (nuclear factor kappa B), activation of anti‐inflammatory transcription factors, such as PPARγ (peroxisome proliferator‐activated receptor gamma), and binding to the GPCR120 (G protein‐coupled receptor 120) (Calder, 2012, 2013; Rogero & Calder, 2018). Furthermore, the enzymatic oxidation of EPA and DHA at the site of inflammation leads to the synthesis of specialized proresolving mediators (SPMs), such as resolvins, protectins, and maresins. These chemicals impede neutrophil transendothelial migration and the generation of cytokines (TNF‐α and IL‐1β) and chemokines through the cyclooxygenase (COX) and lipoxygenase (LOX) pathways (Calder, 2013). Hence, multiple mechanisms come into play to coordinate the reduction of inflammation and assist healing, especially in the respiratory tract. In addition, these SPMs have been demonstrated in numerous studies using animal models for prevention and treatment of acute lung injury (ALI) and ARDS (Gao et al., 2017; Sekheri et al., 2020; Sham et al., 2018; Wang et al., 2018; Zhang et al., 2019). Several human trials have also shown health benefits of DHA and EPA in ARDS‐affected patients. Moreover, according to a recently published Cochrane review, ARDS patients who received EPA and DHA supplements displayed a dramatic improvement in blood oxygenation along with significant decrease in organ failures, the need for ventilation, intensive care unit (ICU) stay, and mortality after 28 days (Dushianthan et al., 2019). All these functions of ω‐3 fatty acids could be very useful in the context of COVID‐19, which is manifested by uncontrolled inflammation (CSS) linked with ALI and ARDS. Taken together, these findings suggest that EPA and DHA may aid in the recovery of SARS‐CoV‐2 patients by reducing inflammation and lung injury, possibly via conversion to SPMs.

#### 4.2.3 Anticoagulation properties

Another complication that commonly exists in severe cases of COVID‐19 is known as coagulopathy. It seems to be associated with occurrence of venous and arterial thromboembolic disease and mimics other systemic coagulopathies that are regularly seen in severe infections, most notably disseminated intravascular coagulation (DIC) (Levi & Iba, 2021). Although supplementation of DHA and EPA has been shown to reduce platelet aggregation and activation in healthy subjects, a higher recommended dose is required in some prothrombotic conditions (Adili et al., 2018). However, clinical efficacy of ω‐3 fatty acids as an antiplatelet therapy in the treatment of COVID‐19 still remains to be validated.

## 5 MILK VITAMINS

The vitamin concentration of AM varies among different animals, and it depends on various factors including species, breed, type of diet and feeding behavior, stage of lactation, health status of the udder, milk and fat yields, and genetic traits of animals. Buffalo milk is 10‐fold richer in vitamin B_6_ and 2‐fold richer in vitamin B_3_ and vitamin E, but markedly poorer in vitamins B_2_, B_6_, and B_9_ than cow milk (Medhammar et al., 2012). Similarly, ewe milk is rich in vitamins (especially vitamin A) than that of other ruminants (especially cows). It is also richer in vitamins B_1_, B_2_, B_3_, B_5_, B_6_, B_12_, folic acid, and vitamin C than goat milk (Park et al., 2007). Levels of folate and Vitamin B_12_ in cow milk are five times higher than those in goat milk. Among different animal species, Dromedary milk is especially rich in vitamin C and vitamin A, which is of special interest in human nutrition (Graulet, 2014). During COVID‐19, vitamins play only symptomatic and supportive roles along with the principle therapy.

### 5.1 Fat‐soluble vitamins

#### 5.1.1 Vitamin A

Vitamin A belongs to the family of retinyl esters, and acts as T cell effector, facilitating adaptive and innate immunity (Kumar et al., 2021). It plays crucial role in immunoregulation along with proliferation and differentiation of T‐lymphocytes into regulatory T cells (Jovic et al., 2020). Retinoic acid (RA), a metabolite of vitamin A (retinol), has been implicated in the modulation of ARDS by influencing the production of IL1‐β and IL‐1 receptor antagonist by alveolar macrophages, and the subsequent pulmonary infiltration of neutrophils (Hashimoto et al., 1998). According to a stereological analysis, it has been observed that RA along with simvastatin improved the injured pulmonary microenvironment and dynamics of lung tissues in the functional repair of respiratory tract (Yang et al., 2015). These findings suggest that vitamin A‐dependent processes may play a role in oxidative damage and/or lung regeneration. According to Sarohan (2020), depletion of RA alters the immune system's shift to the NF‐κB arm, which leads to an excessive release of cytokines, and thus creates CSS as observed in systemic inflammatory response syndrome (SIRS), ARDS, and COVID‐19. As per another scientific investigation, vitamin A present in cow milk may impact lymphocytes homing to the upper part of respiratory system by causing them to produce the tissue homing‐linked markers α4β7 (Perdijk et al., 2018). Additionally, vitamin A was recommended as an alternative CoV therapy and a way to prevent lung infections (Zhang & Liu, 2020). Jee et al. (2013) documented that inadequate vitamin A intake by feedlot calves hampered their antibody response when injected with inactivated BCoV vaccine. As a result, vitamin A may assure a supporting function in the treatment of COVID‐19 together with the creation of good antibody response in people vaccinated with CoV vaccine. Perusal of Table 7 revealed that one cup (244 g) of buffalo and goat milk can meet approximately 50% requirement of vitamin A for adult humans.

**TABLE 7:** Concentrations of vitamins and minerals in milk from different species and estimated adult human requirements fulfilled by one serving (1 cup = 244 g) of milk.

	Concentration in milk from various animal species (μg/100 g)					Estimated requirement for adults (19–65 years) (μg/person/day)^d^ ^,^ ^e^	Approximate % of requirement fulfilled by one serving cup (244 g) of milk				
Vitamins	Cow^a^	Buffalo^b^	Sheep^a^	Goat^a^	Camel^c^		Cow	Buffalo	Sheep	Goat	Camel
A	37.8	53	43.8	55.5	15	270–300	34–31	48–43	40–36	50–45	14–12
D	0.05	ND	0.18	0.0575	ND	5–10	2–1	‐	8.8–4.4	2.8–1.4	‐
E	60	ND	ND	70	ND	7500–10,000	2–1.5	‐	‐	2.3–1.7	‐
K	0.2	ND	ND	0.3	ND	55–65	0.9–0.8	‐	‐	1.3–1.1	‐
B_1_	45	52	80	68	33	1100–1200	10–9	12–11	18–16	15–14	7
B_2_	160	135	376	210	42	1100–1300	35–30	30–25	83–71	47–39	9–8
B_3_	80	91	416	270	460	14,000–16,000	1.4–1.2	1.6–1.4	7.3–6.3	4.7–4.1	8–7
B_6_	42	23	80	46	52	1300–1700	8–6	4–3	15–11	8.6–6.6	10–7
B_9_	5	6	5	1	0.4	400	3	3.7	3	0.6	0.2
B_12_	0.357	0.36	0.712	0.065	0.2	2.4	36	37	72	6.6	20
C	1940	2300	4160	1290	2400	45,000	11	12	23	7	13
Minerals											
Ca	122,000	112,000	193,000	134,000	106,000	1,000,000–1,300,000	30–23	27–21	47–36	33–25	26–20
P	119,000	99,000	158,000	121,000	63,000	700,000	42	35	55	42	22
Mg	12,000	8000	18,000	16,000	12,000	220,000–260,000	13–11	9–8	20–17	18–15	13–11
Na	58,000	35,000	44,000	41,000	69,000	1,300,000–1,500,000	11–9	7–6	8–7	8–7	13–11
K	152,000	92,000	136,000	181,000	156,000	3,510,000	11	6	9	13	11
Zn	530	410	570	560	440	3000–4200	43–31	33–24	46–33	46–33	36–26
Se	0.96	ND	1.00	1.33	ND	26–34	9–7	‐	9–7	12–10	‐
I	2.1	ND	20	22	ND	150	3	‐	33	36	‐
Cu	60	35	40	50	160	1200	12	7	8	10	33

^a^ Park et al. (2007).

^b^ Patino et al. (2007).

^c^ Sawaya et al. (1984).

^d^ WHO (2004).

^e^ WHO (2012).

#### 5.1.2 Vitamin D

Vitamin D helps in the absorption of calcium and phosphorus and maintains bone homeostasis. In addition, its metabolites strongly influence immunity via differentiation of monocytes to macrophages and increasing their killing capacity; modulating the production of inflammatory cytokines; and supporting antigen presentation (BourBour et al., 2020). Many immune cells (B cells, T cells, and antigen‐presenting cells) possess vitamin D receptors that affect their function after ligand binding, and thus it has capability of acting in an autocrine manner in a local immunologic milieu (Aranow, 2011). Furthermore, its metabolites appear to control the synthesis of particular antimicrobial proteins for direct killing of pathogens, and as a result are probably helpful in lowering lung infections (Calder et al., 2020). Several studies demonstrated the role of vitamin D in reducing the risk of acute viral respiratory tract infections and pneumonia (Laplana et al., 2018; Teymoori‐Rad et al., 2019). The possible mechanisms of action might be due to inhibition of viral replication or anti‐inflammatory or immunomodulatory roles (Ali, 2020). The SARS‐CoV‐2 target ACE2 has been revealed to be affected by vitamin D also. So, by activating ACE2, vitamin D may reduce ARDS and ALI brought on by SARS‐CoV‐2 (Xiao et al., 2021). Ilie et al. (2020) discovered an adverse relationship between vitamin D status with morbidity and mortality of COVID‐19 in some countries including Italy, Turkey, Spain, Ireland, Slovakia, Norway, Germany, Estonia, Hungary, Portugal, Finland, United Kingdom, France, Iceland, Sweden, Denmark, Belgium, Switzerland, and the Czech Republic. Studies have shown that vitamin D decreases the overexpression of proinflammatory cytokines (TNF‐α, IL‐1α, IL‐1β, and IFN‐γ), boosts the expression of anti‐inflammatory cytokines (Hughes & Norton, 2009), and releases defensins and cathelicidins that stop viral replication (Grant et al., 2020), potentially speeding up the recovery of patients with viral infections, particularly COVID‐19 (Balla et al., 2020). Therefore, vitamin D could be a promising option for the treatment of this novel CoV due to its antimicrobial and antioxidative effects and helping the immune system against lung infection and airway inflammation. Although one cup of sheep milk provides comparatively higher amount of vitamin D than the other species' milk, it contributes only about 9% of requirement for adult humans (Table 7).

#### 5.1.3 Vitamin E

Vitamin E is a fat‐soluble antioxidant that scavenges free radicals, ROS, and reactive nitrogen species (RNS) by donating hydrogen ions from its chromanol ring (Gulcin, 2020). Thus, it decreases oxidative stress, which is mainly responsible for causing ARDS including COVID‐19 (Chernyak et al., 2020). Deficiency of vitamin E leads to ferroptosis, which in turn is accounted for one of the central death mechanisms in COVID‐19 patients due to multiple damages to the heart, liver, lungs, kidneys, gut, and nervous system (Ashrafizadeh et al., 2019). Consumption of vitamin E at high dose (500 mg/kg) may prevent ferroptosis in COVID‐19 patients by inhibiting lipoxygenase and peroxyl radicals (Tavakol & Seifalian, 2022). Vitamin E also causes immune stimulation in animal and human models via three pathways—(1) reducing the production of NO, and thereby PGE2 along with suppression of cyclooxygenase‐2, (2) modulating the Th1/Th2 balance, and (3) initiation of T cell signals (Lee & Han, 2018). The vitamin E concentration in bovine milk is approximately 0.312 mg/g (Arora et al., 2022), and human requirement is about 12–15 mg/day/person (Insel et al., 2004). The consumption of one cup of either cow or goat milk will provide only 2% of the total need for vitamin E for adult humans (Table 7).

#### 5.1.4 Vitamin K

Vitamin K is a fat‐soluble vitamin that is normally required for posttranslational chemical modification in a small group of proteins with calcium‐binding properties. These are vitamin K‐dependent proteins, also known as Gla proteins or coagulation proteins. These proteins are synthesized in the liver and comprise factors II, VII, IX, and X, which have hemostatic role, and proteins C and S, which have an anticoagulant role. Thus, vitamin K plays important roles in coagulation and possibly also in lung diseases (Dofferhoff et al., 2021). Vitamin K possesses a key role in the pathology of COVID‐19, and its deficiency is associated with CSS, thrombotic complications, multiple organ damage, and high mortality (Ali et al., 2021). It has been documented that deficiency of vitamin K during the early phase of COVID‐19 infection may contribute to the activation of the Th2 storm with increased production of IL‐6 (Anastasi et al., 2020). Vitamin K exerts its anti‐inflammatory action via reduction of prostaglandin E2 (PGE‐2), COX‐2, and IL‐6 (Suleiman et al., 2013). Thus, reduced vitamin K status acts as a potentially modifiable prognostic risk factor in COVID‐19. Although we cannot get sufficient amounts of vitamin K from AM (Table 7), it may be advantageous when considered in conjunction with the effects of other AM constituents that boost the immune system and play protective role.

### 5.2 Water‐soluble vitamins

#### 5.2.1 Vitamins B

Eight different vitamins, including B_1_, B_2_, B_3_, B_5_, B_6_, B_7_, B_9_, and B_12_, make up the vitamin B complex. Their concentration in AM varies, and they are crucial to cell metabolism. While working on macrophages, vitamin B1 may have an anti‐inflammatory impact and reduce NF‐κB activation induced by oxidative stress (Spinas et al., 2015). It significantly contributes to the eradication of SARS‐CoV‐2 by fostering humoral and cell‐mediated immunity in COVID‐19 patients (Shakoor et al., 2021). A score‐matched investigation was carried out in 738 critically ill COVID‐19 patients, and it was observed that patients who received thiamine as an adjunctive therapy were less likely to have thrombosis during ICU stay by 81% along with significantly lower in‐hospital mortality by 51% (Al Sulaiman et al., 2021). Thiamine has also been reported to act as attenuator of Th17‐mediated IL‐17 proinflammatory response (CSS) and subsequent neurological symptoms observed in COVID‐19 patients (Vatsalya et al., 2020). According to a study, it has been documented that riboflavin and UV light have ability to reduce the titer of SARS‐CoV‐2 to the limit of detection in human plasma and whole blood (Ragan et al., 2020). This could provide broad‐spectrum safety for use of pathogen‐reduced blood products in critically ill patients with COVID‐19. Vitamin B_3_ has been found to decrease IL‐6, IL‐1β, and TNF‐α in stimulated alveolar macrophages (Kumar et al., 2021). Moreover, in COVID‐19 patients, IL‐6 targeting could reduce inflammation (Liu, Li, et al., 2020). Deficiency of vitamin B_6_ is linked with lower immune function and higher susceptibility to viral infections (Rail & Meydani, 1993). Vitamin B_6_ may exert a protective effect in ameliorating the severity of COVID‐19 and its complications (hypertension, cardiovascular disease, and diabetes) by suppressing inflammation (CSS), inflammasomes, oxidative stress, and carbonyl stress, regulation of Ca^2+^ influx, elevation of carnosine (a cardioprotector), and immune function improvement (Kumrungsee et al., 2020). According to another study, vitamin B_9_ (folic acid) may help to prevent or alleviate respiratory involvement in early stages via inhibition of furin activity in COVID‐19 patients (Sheybani et al., 2020). The SARS‐CoV‐2 protein M‐pro's crystal structure was used in a molecular docking study to evaluate therapeutic compounds that could be used to treat COVID‐19. In order to forecast the binding, docking scores, lipophilic and hydrogen‐bonding interactions, and ligand proficiency parameters were used, and it was observed that vitamin B_12_ and nicotinamide occupied fourth and sixth positions, respectively (Kandeel & Al‐Nazawi, 2020). Research shows that vitamin B_12_ possesses strong affinity for binding to SARS‐CoV‐2 protease (Kandeel & Al‐Nazawi, 2020). Furthermore, deficiency of vitamin B_12_ causes symptoms (i.e., increased oxidative stress, homocysteine concentration, thrombocytopenia, increased lactate dehydrogenase, low reticulocyte count, DIC, vasoconstriction, and renal and pulmonary vasculopathies) similar to that of COVID‐19 (dos Santos, 2020). Thus, vitamin B_12_ may serve as an attenuator to COVID‐19 symptoms. Among different animal species, sheep milk is rich in vitamin B complex and just one cup of it provides about 83% of vitamin B_2_, 72% of vitamin B_12_, 18% of vitamin B_1_, 15% of vitamin B_6_, and 7% of vitamin B_3_ requirements needed for adult humans (Table 7).

#### 5.2.2 Vitamin C

Vitamin C possesses antiviral characteristics that include alleviating endothelial dysfunction, boosting and regulating the production of IFN‐α and cytokines, decreasing inflammation, and reestablishing mitochondrial function (Carr & Maggini, 2017). According to reports, vitamin C administration lowers the incidence of sepsis and ARDS as well as other upper respiratory tract infections (Kashiouris et al., 2020). A study in adult patients revealed that two doses of vitamin C reduced the length of pneumonia in a dose‐dependent manner (Baladia et al., 2020). Another problem associated with COVID‐19 is managing a number of patients at the same time in ICUs. According to a meta‐analysis of 12 studies including 1766 ICU patients, vitamin C shortens patients' stays there by 8% (Hemila & Chalker, 2019). In cow milk, the approximate concentration of vitamin C is 5.98 mg/L (Foroutan et al., 2019), but humans require about 30–45 mg/day/person (WHO, 2004). One cup of sheep milk provides just 23% of vitamin C requirement for adult humans (Table 7).

From the above discussion, it is clear that although the concentration of vitamins in AM is low, these can be used as adjuvants in addition to COVID‐19's principle therapy.

## 6 MILK MINERALS

Milk is a rich source of minerals including macro (Ca, P, K, Na, and Mg) and micro (Zn, Cu, Se, and I) minerals. However, their concentration varies considerably (Table 7) and depends on various factors, namely species, breed, diet, individual animal, stage of lactation, and status of udder health (Park & Chukwu, 1988). Among different AMs, overall sheep and goat milk possess comparatively higher Ca, P, Mg, Zn, Se, and I and less Na than cow milk (Table 7). The minerals which could play important roles in prevention and/or treatment of COVID‐19 are briefly discussed below.

### 6.1 Macrominerals

#### 6.1.1 Calcium

Calcium plays important role in normal respiratory functioning, energy generation, immunity strength, nerve conduction, blood coagulation, regulating heart rate, secretion of hormones, enzymes, and contraction of muscles (Bailey et al., 2011). Many scientists have reported a relatively high prevalence of hypocalcemia in COVID‐19 patients (di Filippo et al., 2022). In North America, it was reported that 60% of SARS patients were suffering from hypocalcemia at the time of hospital admission, and about 70% suffered during hospitalization (Booth et al., 2003). Hypocalcemia and low calcium levels are strongly correlated with a more pronounced inflammatory response in COVID‐19 patients (di Filippo et al., 2022). Different studies reported that there was a strong negative correlation between calcium levels and C‐reactive protein (CRP), procalcitonin (PCT), IL‐6, and D‐dimer, but positive correlation with lymphocyte count (Liu, Han, et al., 2020; Sun et al., 2020; Tomic et al., 2021). Sun et al. (2020) observed worst clinical findings (multiple organ dysfunction syndrome, septic shock and mortality) in patients with low‐serum calcium levels (especially ≤2.0 mmol/L) suffering from COVID‐19. Also according to a case–cohort research study, serum Ca was proved to be inversely associated with the risk of ischemic stroke (Dibaba et al., 2019). Thus, correcting calcium imbalance by taking a daily calcium supplement can avoid organ injury in the early stage of patients suffering from mild/moderate COVID‐19, but it needs further clinical research. Among domestic AM, one cup of commonly available cow and buffalo milk can meet approximately 30% of calcium requirement for adult humans (Table 7). However, same quantity of sheep milk can meet nearly 50% requirement of calcium for adult males.

#### 6.1.2 Phosphorus

Phosphorus is involved in making proteins required for growth and maintenance, and repair of cells and tissues (Vance, 2011). It has been demonstrated that the immune function of old‐age patients with severe pneumonia and/or hypophosphatemia is significantly lower than that of their healthy counterparts, and the patients with hypophosphatemia tend to show a state of immunosuppression (Xuekai et al., 2019). Hypophosphatemia is a condition, commonly seen in ICU patients suffering from metabolic or respiratory alkalosis, diabetic ketoacidosis, and alcoholism (Koumakis et al., 2021). According to a study, the incidence of hypophosphatemia in critically ill patients can be as high as 44.8% (Zazzo et al., 1995). Thus, serum P levels could serve as a reference index to determine the success of treatment in patients with acute exacerbations of chronic obstructive pulmonary disease (Zhao et al., 2016). Since hypophosphatemia is positively correlated with COVID‐19 severity, it is clinically important to increase the monitoring of serum phosphorus levels in COVID‐19 patients who are severely or critically ill so as to promptly cure hypophosphatemia in order to raise the rate of prognosis (Xue et al., 2020). However, more detailed scientific investigation is required to demonstrate the advantage of reinstating hypophosphatemia in patients suffering from COVID‐19. As evident from Table 7, consumption of just one cup of AM can meet up to 50% requirement of P for adult humans.

#### 6.1.3 Magnesium

Mg plays important role in human body. In addition to its physiological roles (normal metabolism, transport of potassium ion or calcium ion, etc.), it acts as anti‐inflammatory (Turner et al., 2017), anti‐oxidative (Guzel et al., 2018), anti‐spasmodic (Yen & Thwaites, 2019), vasodilatory (Wang et al., 2019), and neuroprotective (Jameson & Bernstein, 2019). Thus, Mg plays vital role in maintenance of normal human health by regulating reproductive, cardiovascular, digestive, nervous, and respiratory systems. Mg possesses a strong relationship with both specific and nonspecific immune responses and deficiency of the same may cause impaired cellular and humoral immune functions (Laires & Monteiro, 2008). Low levels of Mg activate inflammation by sensitizing sentinel cells to the noxious agent, priming phagocytes, and promoting a cascade of vascular and cellular events that characterize the process (Castiglioni et al., 2017). Experimental evidence shows that Mg deficiency may cause activation of leukocytes and macrophages, the release of proinflammatory molecules such as IL‐1, IL‐6, TNF‐α, vascular cell adhesion molecule‐1, plasminogen activator inhibitor‐1, and excessive production of free radicals (Nielsen, 2018). Thus, subclinical Mg deficiency exacerbates virus‐induced inflammation along with uncontrolled release of proinflammatory cytokines, resulting in CSS. Keeping the above facts in mind, it can be said that supplementation of adequate Mg in diet may play important roles in fight against COVID‐19 by supporting the immune system, suppressing the release of some proteins (NF‐κB, IL‐6, CRP, etc.), regulating renal potassium loss, and activating and enhancing the functionality of vitamin D (Wallace, 2020). Table 7 shows that commonly available cow and buffalo milk can meet only 10%–13% of human requirements of Mg, which could be beneficial along with synergistic effects of other milk ingredients. However, one cup of sheep milk can meet up to 20% of Mg requirement for adults.

#### 6.1.4 Sodium

Sodium plays important role in the maintenance of normal cellular homeostasis in addition to the regulation of fluid and electrolyte balance. Studies in rats indicated that high dietary sodium intake results in downregulation of the ACE2 expression in kidney tissue (Berger et al., 2015; Cao et al., 2017). According to a report, hyponatremia was much common (50%) among hospitalized COVID‐19 patients in the United States (Aggarwal et al., 2020). Also, it was observed that lower levels of serum sodium concentration resulted in higher IL‐6 production along with a more severe outcome of COVID‐19 disease (Berni et al., 2020). Thus, sodium shows a considerable impact on the therapeutic outcomes of patients with COVID‐19. Hence, it is proposed to monitor sodium intake level of patients during severe COVID‐19 infections and low sodium intake must be corrected in early stages (Post et al., 2020). However, further clinical research is required to be conducted in this direction since a potential conflict regarding sodium intake in patients with hypertension, diabetes, and kidney disease exists. Commonly available cow milk (one cup) can meet about 11% requirement of sodium for adults (Table 7). Thus, positive effects of sodium along with other ingredients can be observed by consumption of AM.

#### 6.1.5 Potassium

Hypokalemia (low level of potassium) may increase the risk of ARDS and acute cardiac damage, which are thought to be the most frequent complications of COVID‐19. According to a study, hypokalemia was seen in 41% of non‐ICU admitted patients suffering from severe infection of COVID‐19 (Alfano, Ferrari, et al., 2021). Hypokalemia has been mentioned as a potential manifestation of COVID‐19, probably due to the interaction of SARS‐CoV‐2 with the renin–angiotensin–aldosterone system (Alfano, Guaraldi, et al., 2021). However, the etiological mechanisms responsible for the development of hypokalemia in COVID‐19 need to be documented convincingly. The higher prevalence of hypokalemia is associated with the requirement for invasive mechanical ventilation among COVID‐19 patients (Moreno‐Perez et al., 2020). Hence, it appears to be a sensitive biomarker for the progression of severity of COVID‐19. Consumption of one cup of cow milk can meet 11% requirement of K recommended for adults (Table 7).

### 6.2 Microminerals

#### 6.2.1 Zinc

Zinc is the second most abundant trace metal in the human body, and plays critical roles in immune homeostasis, inflammation, and antiviral immunity (Pal et al., 2021). It is essential for the functioning and proliferation of neutrophils, macrophages, T and B lymphocytes, and NK cells (Rahman & Idid, 2021). In addition, it also impairs the replication of SARS‐CoV through alteration of RdRp activity by directly affecting the template binding (Te Velthuis et al., 2010). Zinc also suppresses the anti‐inflammatory activity by reducing the secretion of proinflammatory cytokines such as IL‐6, and monocyte signal transduction, thus protecting from severe lung injury due to CSS in COVID‐19 patients (Mayor‐Ibarguren & Robles‐Marhuenda, 2020). It exhibits antioxidant role by inhibiting the production of ROS, such as superoxide anion, H_2_O_2_, and radical hydroxyl as well as RNS including peroxynitrite (Hadwan et al., 2014; Ogawa et al., 2011). In antioxidant proteins, Zn ion is associated with its binding to thiol groups, thus protecting them from oxidation (Olechnowicz et al., 2018). According to WHO (2003), about one‐third of total world's population is affected by zinc deficiency, which is responsible for 16% of all deep respiratory infections worldwide. This gives a strong indication of association of zinc deficiency with the risk of infection and severe progression of COVID‐19, and suggests potential benefits of zinc supplementation. In nutshell, zinc improves mucociliary clearance, strengthens the integrity of the epithelium, enhances antiviral immunity, reduces viral replication and hyper‐inflammation, supports antioxidative effects, and thus reduces lung damage (Wessels et al., 2020). This may have potential benefits for old‐age patients suffering from COVID‐19 along with comorbidities. From all these facts, it seems that zinc supplementation may be useful for the prevention and treatment of COVID‐19. Among the milk of different species, maximum requirement (33–46%) can be met with one cup of sheep or goat milk (Table 7). Therefore, positive effects of zinc on the immune system and disease resistance can be seen with the consumption of domestic AM.

#### 6.2.2 Selenium

Selenium is an essential trace element required for the functioning of all organisms. The very first evidence of a link between Se status and susceptibility of humans to a viral infection came from the investigation of cardiomyopathy (Keshan disease) among the population in Heilongjiang province in China (Loscalzo, 2014). In addition to supporting T cell‐dependent antibody synthesis, selenium also ensures that T cell maturation and functions are maintained properly (Bae & Kim, 2020). It also enhances the activities of CD4^+^ T cells, NK cells, and B cells thereby boosting the immune system (Chowdhury, 2020). Thus, deficiency of selenium may weaken the immune defense against COVID‐19 and cause progression to severe disease, as described by a study in South Korea (Im et al., 2020). In selenium‐deficient people, immune cells synthesize less selenoproteins, thereby increasing the risk of being infected by SARS‐CoV‐2 with adverse outcomes (Khatiwada & Subedi, 2021). Also, according to Moghaddam et al. (2020), selenium deficiency was associated with higher mortality rate in COVID‐19 patients. This was further confirmed, when in a clinical trial, parenteral administration of selenium reduced illness severity and incidence of hospital‐acquired pneumonia in ICU patients suffering from SIRS (Manzanares et al., 2011).

Selenium and selenoproteins inhibit NF‐κB and decrease viral replication (Hiffler & Rakotoambinina, 2020). Additionally, it has a significant impact on reducing the ROS generated in response to diverse viral infections (Tomo et al., 2021). Formation of blood clots acts as leading cause of death in patients with COVID‐19. Selenium may play positive role by reducing their formation via decreasing the ratio of thromboxane A2 to prostacyclin I2 as demonstrated in rats (Haberland et al., 2001). At physiological levels, selenium also inhibits activation of the NF‐κB transcription factor resulting in decreased production of inflammatory cytokines such as IL‐6 as observed in cell line studies, animal models, and human studies (Maehira et al., 2003). Thus, selenium may act as antiviral, antioxidative, anticoagulant, and immunomodulator in COVID‐19 patients.

From the above findings, it seems that selenium possesses a relevant role in COVID convalescence and supports the discussion on adjuvant Se supplementation in severely diseased and Se‐deficient patients. Although consumption of one cup of AM just fulfills 9%–12% of daily requirement for adult humans (Table 7), it is important when viewed along with synergistic effects of other components.

#### 6.2.3 Iodine

Iodine, an essential element, is crucial for controlling thyroid gland activity and the production of thyroid hormones, which in turn control critical metabolic, brain developmental, and growth processes (Grau et al., 2015). In addition, iodine also acts as an antioxidant, anti‐inflammatory, antiproliferative, and differentiation agent (Boretti & Banik, 2022). Milk and milk products are important sources of iodine (Van der Reijden et al., 2017). Iodine is also present in breast milk as potassium iodide or sodium iodide, and in infant formulas (Pearce et al., 2004). However, the concentration of iodine in AM is highly variable and depends on various factors, such as quantity and type of iodine in feed intake, antinutritional factors, milk yield, milk processing (skimming and heat treatment), and farm management practices including animal keeping, and teat dipping operations (Miklas et al., 2021). Iodine supplementation may augment mucosal antiviral defense. According to a study, it has been demonstrated that iodide when added with LPO enzyme exhibited robust antiviral activity in cultured cells against adenovirus (nonenveloped, dsDNA) and RSV (enveloped, −ve sense ssRNA) (Fischer et al., 2011). Since the mechanism is effective against two different types of viruses, it is most likely to be effective against SARS‐CoV‐2 (enveloped, +ve sense ssRNA) virus also. According to Derscheid et al. (2014), iodine supplementation reduced severity of RSV infection in 3‐week‐old lambs. Moreover, iodine treatment also reduced lung damage and pulmonary expression of RSV antigen in lambs affected with RSV disease. According to an in vitro study, iodide has also been reported to possess immunomodulatory effects on HPBL, suggesting that optimally iodide‐saturated cells could enhance the immune system and improve clearance of infections (Bilal et al., 2017). Iodide also effectively scavenges ROS in human blood cells (Kupper et al., 2008). An in vitro study has shown that iodide has significantly enhanced IgG synthesis by HPBL (Weetman et al., 1983). Iodine as a dietary supplement may limit the side effects (inflammatory processes and toxin removal) of COVID‐19 vaccination, but this area further needs research (Boretti & Banik, 2022). Among AM, one cup of cow and goat milk can meet about 3% and 36% of daily requirements for adult humans, respectively (Table 7). Thus, when dairy cows are properly supplemented with mineral premixes and licks, favorable benefits of iodine on the immune system can be shown through milk consumption.

#### 6.2.4 Copper

Copper, a necessary micromineral for all species, plays an active role in immunological functions, free‐radical protection, and respiration (Fooladi et al., 2020). It helps in the functioning of T helper cells, B cells, neutrophils, NK cells, and macrophages in order to kill infectious microbes, and enhances cell‐mediated immunity and production of specific antibodies (Raha et al., 2020). Moreover, copper regulates the level of IL‐2 which is critical in T helper cell proliferation, the balance between Th1 and Th2 cells, and NK cell cytotoxicity which is also important in management of immune dysregulation in critically ill COVID‐19 patients (Hopkins & Failla, 1997). Cu may also suppress the production of inflammatory cytokines, chemokines, and adhesion molecules by downregulating the expression of NF‐*κ*B, which is generally activated by virus‐induced ROS (Rani et al., 2021). This could be highly useful during CSS phase of COVID‐19. As an antioxidative molecule, it may stimulate stress‐signaling pathways including antiapoptotic phosphoinositide‐3‐kinase/Akt cascade and may stabilize proteins, so as to make them less prone to oxidation (Klotz et al., 2003). Copper is also required for enzymes such as CuZn‐superoxide dismutase which catalyzes the dismutation of superoxide to oxygen and H_2_O_2_. These peroxides are then subsequently reduced by the selenoenzyme GSH peroxidase (GPx), thereby protecting mammalian cells against oxidative damage (Uriu‐Adams & Keen, 2005).

As an antiviral molecule, it may inactivate RNA viruses by destroying their viral genomes and/or blocking the activity of papain‐like protease‐2, a protein that SARS‐CoV‐1 requires for replication (Raha et al., 2020). During the process of apoptosis, copper is also involved in the formation of autophagic vacuoles, thereby contributing to the cell's antiviral defense (Fooladi et al., 2020). Based on the above facts, it seems that enrichment of plasma copper levels will boost both innate and adaptive immunity in people. Moreover, owing to its potent antiviral activities, Cu may also act as a preventive and therapeutic regime against COVID‐19. Among the domestic AM, camel milk contains maximum concentration of copper (Table 7). However, one cup of commonly available cow milk can only meet 12% of daily human requirement for adults. Although drinking milk alone does not provide adequate amounts of copper to significantly boost the immune system, looking at the positive effects of these metabolites as a package, and the role and contribution of each in achieving the ultimate goal can be significant.

## 7 CONCLUSION

**FIGURE 2:**
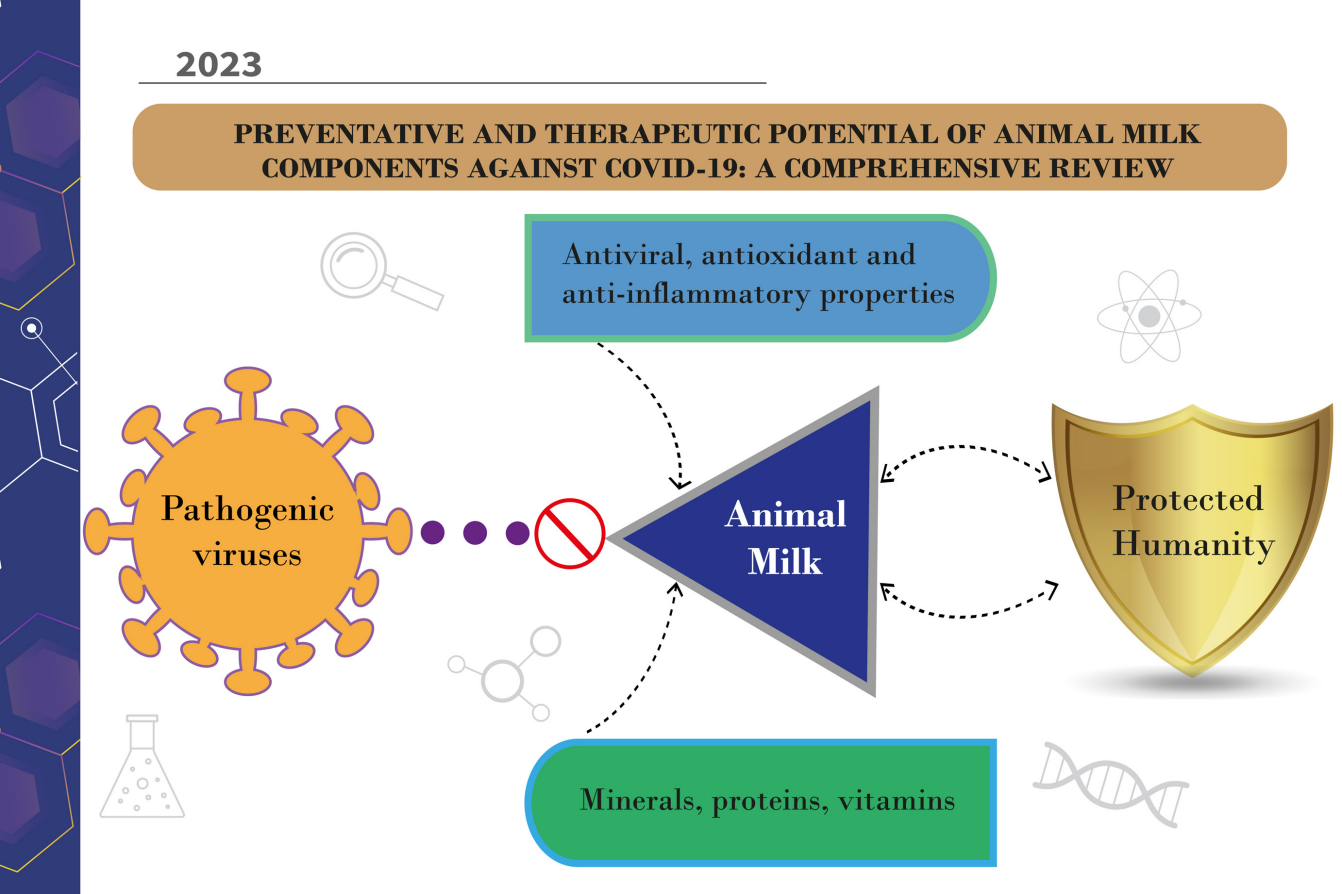
Therapeutics properties of animal milk components against pathogenic viruses such as COVID‐19.

A substantial global decline in human well‐being and health maintenance as well as a significant negative effect on healthcare delivery systems have been brought about by the COVID‐19 pandemic. Milk and/or its ingredients may show additive or synergistic effects with each other or some drugs. Some ingredients (e.g., lactoferrin) may provide direct antiviral effects against COVID‐19. Other ingredients may help indirectly either during hyperinflammation (lactoferrin, caseins, lysozyme, and lactoperoxidase) or during coagulopathy disorders (e.g., caseins). Also, success of this type of strategy and tactics can amplify research and explore the intrinsic genetic well‐being potential that food and its contents bring. Given the intricacy of the condition and the current dearth of new pharmaceutical treatments, these findings may be incorporated into policies or suggestions for managing COVID‐19 as complementary methods to enhance patients' rehabilitation, and therefore amplify the odds against virus and other pathogens, by taking advantage of the qualities within food. These preventative and therapeutic properties of animal milk components are depicted in Figure 2.

## FUNDING INFORMATION

The authors reported there is no funding associated with the work featured in this article.

## CONFLICT OF INTEREST STATEMENT

The authors declare that they have no financial conflict of interest or personal relationships that influence the work described in this paper.

## ETHICAL APPROVAL

This study involved no human or animal subjects.

## Data Availability

Data sharing is not applicable to this article as no new data were created or analyzed in this study.
